# Mechanistic within-host models of the asexual *Plasmodium falciparum* infection: a review and analytical assessment

**DOI:** 10.1186/s12936-021-03813-z

**Published:** 2021-07-10

**Authors:** Flavia Camponovo, Tamsin E. Lee, Jonathan R. Russell, Lydia Burgert, Jaline Gerardin, Melissa A. Penny

**Affiliations:** 1 Swiss Tropical and Public Health Institute, Basel, Switzerland; 2 University of Basel, Basel, Switzerland; 3 Center for Communicable Disease Dynamics, Department of Epidemiology, Harvard T. H. Chan School of Public Health, Boston, MA 02115 USA; 4 Institute of Disease Modeling, Bill & Melinda Gates Foundation, 500 5th Ave N, Seattle, WA 98109 USA; 5 Department of Preventive Medicine and Institute for Global Health, Northwestern University, Chicago, IL USA

**Keywords:** *Plasmodium falciparum*, Within-host mathematical models, Asexual parasite dynamics

## Abstract

**Background:**

Malaria blood-stage infection length and intensity are important drivers of disease and transmission; however, the underlying mechanisms of parasite growth and the host’s immune response during infection remain largely unknown. Over the last 30 years, several mechanistic mathematical models of malaria parasite within-host dynamics have been published and used in malaria transmission models.

**Methods:**

Mechanistic within-host models of parasite dynamics were identified through a review of published literature. For a subset of these, model code was reproduced and descriptive statistics compared between the models using fitted data. Through simulation and model analysis, key features of the models were compared, including assumptions on growth, immune response components, variant switching mechanisms, and inter-individual variability.

**Results:**

The assessed within-host malaria models generally replicate infection dynamics in malaria-naïve individuals. However, there are substantial differences between the model dynamics after disease onset, and models do not always reproduce late infection parasitaemia data used for calibration of the within host infections. Models have attempted to capture the considerable variability in parasite dynamics between individuals by including stochastic parasite multiplication rates; variant switching dynamics leading to immune escape; variable effects of the host immune responses; or via probabilistic events. For models that capture realistic length of infections, model representations of innate immunity explain early peaks in infection density that cause clinical symptoms, and model representations of antibody immune responses control the length of infection. Models differed in their assumptions concerning variant switching dynamics, reflecting uncertainty in the underlying mechanisms of variant switching revealed by recent clinical data during early infection. Overall, given the scarce availability of the biological evidence there is limited support for complex models.

**Conclusions:**

This study suggests that much of the inter-individual variability observed in clinical malaria infections has traditionally been attributed in models to random variability, rather than mechanistic disease dynamics. Thus, it is proposed that newly developed models should assume simple immune dynamics that minimally capture mechanistic understandings and avoid over-parameterization and large stochasticity which inaccurately represent unknown disease mechanisms.

**Supplementary Information:**

The online version contains supplementary material available at 10.1186/s12936-021-03813-z.

## Background

The complex life cycle of *Plasmodium falciparum* involves many parasite stages within both the mosquito and human host. *Plasmodium falciparum* induces complex and non-sterilizing immune responses with repeated possible exposure in both the human host and mosquito. The human blood-stage infection plays a crucial role in both disease burden and transmission. Indeed, the length and magnitude of asexual parasite infection both drive clinical symptoms within a host and the transmission potential through the level of gametocytes. Thus, understanding within-host dynamics of the asexual parasite stage is essential for both the development of drugs or other tools that target asexual or gametocytes stages, and to assess burden or transmission dynamics. Within a human host, the *P. falciparum* malaria cycle begins with sporozoites transmitted by infectious mosquito bites that travel from the skin to the liver [1]. Following replication in the liver, merozoites are released into the bloodstream [1, 2]. These merozoites subsequently infect red-blood cells (RBCs) and in vitro*,* approximately 16 merozoites emerge from a single merozoite in a 48 h asexual blood-stage cycle [3]. The blood-stage infection can persist for over 300 days if untreated [4]. A small fraction of asexual parasites convert into gametocytes [2], responsible for the transmission from human to mosquito.

The asexual malaria life cycle drives clinical disease in an individual, with many processes eliciting or evading the immune response [1]. After invasion of a RBC, merozoites are no longer directly exposed to immune actors. However, with hundreds of parasite proteins exported to the erythrocyte’s cell surface, immune effectors can recognize an infected RBC (iRBC) [1]. Naturally acquired immune responses recognize erythrocyte surface antigens of iRBCs or antigens of the free merozoites [1], as well as antigens from liver and sexual stages [5, 6]. Exported parasite proteins on the cell’s surface give the cell the capability to adhere to the blood vessel’s wall, and thus evade splenic clearance [1]. Furthermore, expression of the most characterized exported protein, the erythrocyte membrane protein 1 (PfEMP1), can be switched by the parasites from a large library of variants [1]. New protein conformations are produced to avoid detection, requiring the host to mount new immune responses [1]. *Plasmodium falciparum* escapes the immune response by successively expressing one out of 50–60 different PfEMP1 genes [7]. The switching mechanisms remain uncertain, but switching between the PfEMP1 variants needs to be quick enough to evade the immune system and avoid splenic clearance, while slow enough to avoid variant exhaustion and maintain the chronic nature of the infection [7]. In endemic areas where populations are continuously exposed to malaria, repeated infections lead to acquired immunity, preventing severe cases of malaria and death but without leading to sterilizing protection for infection [1].

Many mathematical and statistical models have been developed to understand population level dynamics of malaria transmission and impact of interventions (reviewed in [8, 9]), or to understand within host dynamics. Although there is a long history of mathematical modeling of malaria parasite within-host dynamics over the years, the substantial biological unknown elements of both parasite and host dynamics and the highly variable nature of infection patterns, make it difficult to assess model accuracy. Furthermore, as there is limited within-host data available for infections from either immunologically naïve or non-naïve individuals, there is no “gold standard” data set or model to compare. In 1999, Molineaux and Dietz reviewed published intra-host models [10], indicating the first within-host model of malaria was likely developed in 1989 by Anderson et al*.* [11]. Anderson and colleagues [11] described parasite dynamics via a set of differential equations representing uninfected RBCs, iRBCs, merozoites, and immune effectors [11]. This model along with the others reviewed led Molineaux and Dietz to conclude that existing models lacked realism and did not make quantitative comparisons to real data. They further concluded that the reviewed models did not allow for inter-individual variability in the outputs, even though a large variation in infection dynamics exists between individuals [10]. In part to address these concerns, a substantial number of mechanistic within-host models, either standalone or used in larger transmission models, have since been developed. Most of these models were initially parameterized to data from naïve patients, but not necessarily to the limited available data from previously exposed individuals.

Several sources of detailed observations of parasite dynamics and densities in naïve patients exist. In the past, malaria infection was induced to generate fever to treat other illnesses. In particular between 1917 and 1963 malaria was used as a therapy to treat patients with tertiary neurosyphilis before the use of penicillin [12]. The most extensive malaria therapy data set was collated between 1940 and 1963 by Collins and Jeffery [12–15]. The published database consists of 318 patients treated at Columbia, South Carolina and the Milledgeville, Georgia laboratories [12]. This data, referred to here as the malariatherapy dataset, includes patients infected with three different strains of *P. falciparum* for neurosyphilis treatment. The data captures daily parasite counts by microscopy of both gametocyte and asexual parasites, and daily fever charts are available for each patient. This data set is the only detailed representation of the entire *P. falciparum* infection in a naïve population. Other malariatherapy data sets exist, for example of *Plasmodium vivax* infection in naïve and non-naïve individuals [16] (not further discussed here).

Over the last decade, many individual-based models of malaria transmission dynamics have been developed (reviewed in [8]); several of these include models of within-host asexual parasite dynamics [17–20]. A recently published paper [21] investigated common biological assumptions made by within-host models, and concluded that current knowledge is insufficient to capture infection lengths and to explain the chronic nature of malaria infections. They further concluded their model was quite sensitive to small changes in the parameters leading to large instabilities in estimated infection lengths [21]. Since asexual parasite dynamics are particularly important for modelling the effect of malaria interventions targeting humans (such as drugs or vaccines), the within-host model assumptions in these models have the potential to drive predictions at the population level, on either disease burden or intervention impact. Thus, with these within host models widely used in public health research, a good understanding of the overall dynamics, the assumptions, the uncertainties, and limitations of the models are key to critically assess and understand the predictions arising from the use of those models.

In this review, within-host models of asexual parasitaemia were analysed and components of the models which drive predicted dynamics were identified. The identified models were used to investigate malaria interventions such as drugs or vaccines, either as stand-alone within-host models or used in combination with transmission models. The review and analytical assessment of each model, including the re-simulation of a subset of models to allow for deeper investigation, provides an overview of the main components of each model and their underlying assumptions. Rather than defining a gold standard, models were discussed on how they differ in their immune responses and parasite growth. This comparison provides an understanding of the benefits and limitations in using these models, which directs and informs future work on within-host models of blood-stage parasitaemia.

## Methods

### Malariatherapy dataset

The malariatherapy dataset is extensively described in the retrospective analysis of Collins et al*.* [12]. Briefly, the dataset consists of parasitaemia and fever records from Columbia, South Carolina, and the Milledgeville, Georgia laboratories of patients with no recorded previous history of malaria infection. Patients were infected by either of three parasite strains (named *McLendon*, *Santee-Cooper*, and *El Limon*), inoculated either by intravenous injection of parasitized blood, bites of infected mosquitoes, or subcutaneous or intravenous injection of sporozoites. Parasitaemia was measured daily via thick and thin peripheral blood films examined microscopically. Detection threshold is defined as 10 parasites/μl. At early stages of the infection, parasitaemia and fever levels were controlled with sub-curative treatment when needed, and in some instances the infection were terminated using drugs.

The dataset reported by Collins et al*.* [22] include 318 patient records, and models used a range of malariatherapy data subsets, to select only infections that cleared naturally and with minimal sub-curative drug administration, and/or select infections considered non-severe. Details on malariatherapy subsets for each model are shown in Table 1 and Additional file 1: Table S2, and the respective publications. The smallest dataset was used for the model by Molineaux et al*.*, which include 35 patient records where infections were not classified as severe, and infections cleared naturally. These included patients inoculated with infected blood (n = 18) and mosquito bites (n = 17), using *El Limon* (n = 34) and *Santee Cooper* (n = 1) strains. In the available dataset, only 315 out of the 318 patient records presented by Collins et al*.* were recovered (missing patient *G-27*, *S-934*, *S-1173*) and the time-series and summary statistics are shown for 315 patients, and 35 patients.

**Table 1:** Overview and main description of the models. The model description includes the main characteristics of variant or total parasite growth, the stochastic components, and the data used for fitting.

General model structure, and parasite dynamics								
Models	Molineaux et al.			Gatton & Cheng	Eckhoff	Childs & Buckee	Gurarie et al.	McKenzie & Bossert
Adapted models		Johnston et al.						
			Challenger et al.					
Publication year	2001	2013	2017	2004	2012	2015	2012	2005
Discrete (2 days time step) or continuous	Discrete			Discrete	Mixed	Discrete	Discrete	Continuous
Patient specific parameters	Yes^*^	No		No	No	No	Yes^**^	Yes^***^
Tracks all parasite variants	Yes	Yes	No	Yes	Yes	Yes	No	No
Number of variants at the start of infection	1		–	5	5	5	–	–
Main assumption on variant switching dynamics	Dependent on variant immune response. Different switching probability for each variant follows geometric distribution		–	Fast and slow switching variants. Independent of immune response, described in [51]	Switching rate per iRBC, thus larger population have higher probability to introduce new variant. Parasites can switch to 7 available variants, out of a total of 50 variants, in each round	Different switching networks are investigated. Network assumed from [33]	Not explicit	–
Assumed multiplication rates	Different for every variant but constant in time (~ $\mathcal{N}\left( {\mu _{m} = 16,~\sigma _{m} = 10.4} \right)$)		(For overall parasites) different for every time step, but correlated to previous time step	16 (constant)	16 (constant)	Variant-dependent but constant in time (~ $\mathcal{N}\left( {\mu _{m} = 16,~\sigma _{m} = 8} \right)$)	Range between 15–50, median 23.91	–
Additional assumptions on growth	–	–	–	–	Dependent on RBC availability	Dependent on RBC availability	Dependent on RBC availability	–
Variability included in the model: stochastic parameters	Multiplication rate, patient specific parameters for the critical densities for innate and general adaptive immune response		Critical densities for innate and general adaptive immune response, and growth rate			Most parameters chosen stochastically (for sensitivity analysis)	Merozoite invasion probability and replication rate, innate and adaptive immune response efficiency and activation threshold, antigenically distinct variant clusters	None
Variability included in the model: probabilistic equations				Effect of immune responses, antibody production, variant switching	Variant switching, effect of variant specific and innate immune response		Falls of immune response due to antigenic switch taken at random	None
Data used for the fitting^a^	35 patients^1^			90 patients^2^	Malariatherapy data^3^	–^4^	122 malariatherapy patients	63 malariatherapy patients^5^

^*^ Patient specific critical densities for innate and general adaptive immune response calculation

^**^ Model calibrated to each of the infection chart separately to generate 50 best parameter sets for each calibrated malariatherapy data set

^***^ Model fitted to each of the infection chart separately, thus all parameters take a patient specific value or are averaged across patients

^1^ *P. falciparum* infections classified as spontaneous cures, out of a total of 334 patients in the malariatherapy data

^2^ Patients from the malariatherapy dataset not treated to modify the primary parasitaemia, and with no curative or subcurative treatment after the primary attack

^3^ Number of patients is not specified. Includes those with high initial peaks and treated during the primary peak

^4^ The model focuses on the effect on outcome of varying the parameters, within biologically reasonable ranges

^5^ Inoculated with *McLendon*. From the patients only the days before any drug or other interventions were used, which included a range between 22 and 159 days of infection

^a^ As described in the initial publications, the models might have been re-fitted when updated, implemented in other research projects, or implemented in transmission models

### Models and simulation code

**Fig. 1:**
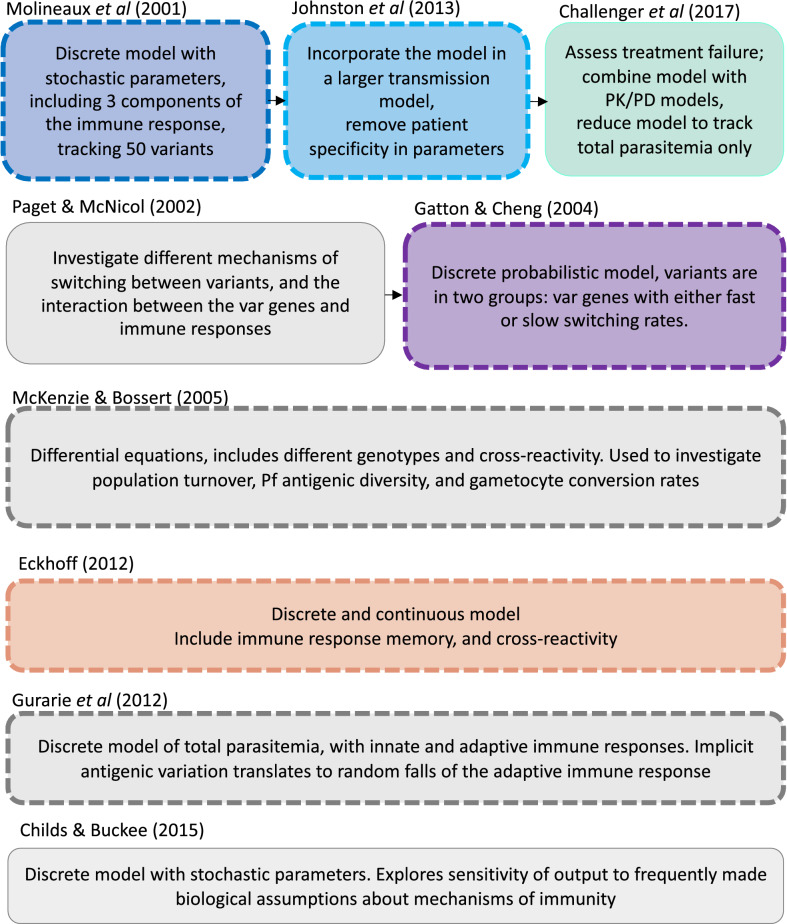
Identified within-host models of asexual parasite blood stage infection. Models are ordered by their publication date on the vertical and horizontal axis, with models adapted from another model displayed on the same row and linked with arrows. Dashed squares represent models that have been used in IBMs according to the systematic review of [6], and colored squares highlight the models that are simulated in this study, where the colours correspond to the results plotted in Figs. 3, 4, 5, 6. Text in each box indicates a brief description of the main features or differences of the models. A more detailed description of the models can be found in the supplementary material or in the source publications [17, 18, 21–26, 31]

Several mechanistic within-host models were identified that are currently implemented in individual-based models (IBMs) from a recently published systematic review of IBMs [8], or used as stand-alone models to understand within host dynamics or assess malaria interventions (drugs or vaccines) targeting humans. The within-host models identified as being part of malaria IBMs are Molineaux et al*.* [22], Johnston et al*.* [23]*,* Gatton and Cheng [24], Eckhoff [19], McKenzie and Bossert [18], and Gurarie et al*.* [17]. Additionally the models of Childs and Buckee [21] and Challenger [25] were included (see Fig. 1, and further details on the models in Results and in the Additional file 1). Five of the eight identified models were re-simulated for a deeper understanding of the underlying dynamics.

For the simulations, the open access code of Johnston et al*.* [23] was ised to recreate the Johnston et al*.* model in Matlab [26]. As both Johnston et al*.* [23] and later model Challenger et al*.* [25] were based on the model of Molineaux et al*.* [22]*,* the code of Johnston et al*.* was used as base code to reproduce the Molineaux et al*.* [22] and the Challenger et al*.* [25] models. Challenger et al*.* provides open access code for their model in C+ + [27], which is used here to validate the model code. The model of Gatton and Cheng [24] was not open access but the model was reproduced in Matlab. The model code for Eckhoff [19] was available from the author’s affiliated groups [28].

### Model simulation and assessment

Each model was simulated 1750 times, which for the patient-specific model of Molineaux et al. corresponds to 50 simulations of the 35 malariatherapy patients used for parameterization. For all other models, this corresponds to 1750 independent realizations of the stochastic models.

Parameter values were fixed to the values defined in their respective publication (note that the parameters are either assumed from literature or fitted in their models, Additional file 1: Table S3). The same stochastic multiplication rate of each PfEMP1 variants was used in the models of Molineaux et al*.* and Johnston et al*.*, except that for Johnston et al. any multiplication rate over 35 was resampled to be within the ranges defined by their model, as defined in their publication. Other models either had a different definition of the parasite’s multiplication rates (Challenger et al*.*) or a constant multiplication rate (Gatton and Cheng, and Eckhoff). Except for Eckhoff, model simulations were undertaken in Matlab [27]. The Eckhoff model was simulated in C+ + [28]. All subsequent analysis of results was performed in R [29]. For the purpose of comparison, all models were simulated for 600 days. Furthermore, the best fit of the model was not selected for each patient and no measurement errors were added (as done by Molineaux et al*.*), as the aim was not to replicate the data but to illustrate the internal behaviour of the models.

Initially models were assessed via nine summary statistics for the malariatherapy data set. These summary statistics were first described in Molineaux et al*.* [22]*,* and were later used as evaluation measures for several of the other models [23–25]. Briefly, the nine summary statistics computed are (i) the slope of the linear regression line from the first positive observed parasitaemia to the first local maximum; (ii) the log_10_ parasite density of the first local maximum, with a local maximum defined as a parasite density greater than the three preceding time steps (t−1 to t−3) and not lower than the three following time steps (t + 1 to t + 3); (iii) the number of local maxima; (iv) the slope of the linear regression through all the log_10_ local maxima; (v–vi) the geometric mean and standard deviation of the geometric means between the local maxima; (vii–viii) the proportion of positive observation in the first half and second half of the interval between the first and last positive observation; and *ix)* the last positive day [22]. A positive parasite density observation was defined as an asexual parasite density equal to, or higher than, 10 iRBC per microlitre, which is aligned with the detection threshold for the malariatherapy dataset [12]. In the Gatton and Cheng model the number of iRBCs is modelled, and thus for consistency and for comparison to other models, the output was converted to iRBC per microlitre assuming a body contains five liters of blood [24].

### Assessment of parasite growth and host immunity for the re-simulated models

In addition to the summary statistics, comparisons were made of estimated overall parasite multiplication rates, the innate, variant specific, general adaptive, and total immune responses, and the subsequent variation across the simulations that reflects individual variation and stochasticity. Parasites are referred to as variant specific to distinguish parasite subpopulation all expressing the same PfEMP1 variant, and thus eliciting the anti-PfEMP1 antibodies specific to that variant. Models were compared by visual inspection of time series plots of these response components to identify the model’s main drivers of parasite density and infection length predictions.

To assess the modelled parasite growth, for the models which use a stochastic and variant-specific multiplication rate, an average inherent parasite multiplication rate (across variants) was defined at each time-step for each model. In Molineaux et al*.* and Johnston et al*.* each variant-specific parasite has its own multiplication rate drawn from a Normal distribution [22, 23]. Therefore, the average multiplication rate of all parasites at each time step is the weighted average of the variant-specific parasite multiplication rates, as follows:

1 $$\bar{M}\left( t \right) = ~\frac{{\mathop \sum \nolimits_{{i = 1}}^{{i = 50}} m_{i} p_{i} \left( t \right)}}{{P_{{tot}} \left( t \right)}},$$

where ${m}_{i}$ is the multiplication rate of variant *i*, ${p}_{i}(t)$ the parasite density of variant *i* at time *t*, and ${P}_{tot}(t)$ the total parasite density at time *t*. For all other models, the overall parasite multiplication is an input parameter and thus does not need to be calculated.

To investigate the modelled immune responses, immune responses were categorized into innate ($${S}_{c}$$), variant-specific ($${S}_{v})$$, and general adaptive ($${S}_{m})$$. Between Molineaux et al*.*, Johnston et al*.,* and Challenger et al*.,* those three terms are directly comparable across models, but for Gatton and Cheng, and Eckhoff the terms are slightly different (see Additional file 1: Table S2). Where the variant specific immune response is tracked for each variant (in Molineaux et al*.,* Johnston et al*.* and Gatton and Cheng), the overall effect of the variant-specific immune response is the weighted average of the variant-specific immune response, as follows:

2 $$\overline{{S_{v} }} \left( t \right) = ~\frac{{\mathop \sum \nolimits_{{i = 1}}^{{i = 50}} S_{{v,i}} \left( t \right)p_{i} \left( t \right)}}{{P_{{tot}} \left( t \right)}},$$

where ${S}_{v,i}(t)$ is the effect of the immune response on variant *i* at time *t*, ${p}_{i}(t)$ the parasite density of variant *i* at time *t*, and ${P}_{tot}(t)$ the total parasite density at time *t*. The innate and general adaptive immune responses are described as an effect on the total parasite density and are reported as such without further modifications. All equations and comparison between the models are summarized in Additional file 1: Table S2.

## Results

### Overall model structure

Via the systematic review of IBMs [8], six mechanistic within-host models were identified as part of transmission models, as represented in Fig. 1, namely Molineaux et al*.* [22], Gatton and Cheng [24], Johnston et al*.* [23], Eckhoff [19], McKenzie and Bossert [18], and Gurarie et al*.* [17]. An additional two models were identified through further literature search, namely Challenger et al*.* [25], and Childs and Buckee [21]. The new quantitative results in the current study focus on five models, namely those of Molineaux et al*.* [22], Gatton and Cheng [24], Johnston et al*.* [23], Challenger et al*.* [25], and Eckhoff [19] (Fig. 1). The models of McKenzie and Bossert [18], Gurarie et al*.* [17], and Childs and Buckee [21] were included in the summary categorizations for comparison.

Most models reviewed here can generally be described in a simple discrete form

$$Y_{t} = m_{t} Y_{{t - 1}} R_{t} ~.$$

Here parasite densities or number of parasites *Y*_t_, at time *t*, depend on the parasite’s multiplication rate ${m}_{t}$ reduced by host effects *R*_*t*_ which can include immune response or RBC resource limitation*.* The immune response can represent up to four components: the innate immune response, and the antibody-driven immune response defined by variant specific, the cross-reactive immune response, and adaptive immune responses, each component including different levels of stochasticity and different functions. The models can be relatively simple and reproduce a smoothed time-course of an infection, such as in McKenzie and Bossert, or include a higher level of complexity to describe more granular parasite dynamics including the typical peaks and throughs observed in clinical data. The latter usually involves explicit or implicit inclusion of a range of variant-specific parasites.

**Fig. 2:**
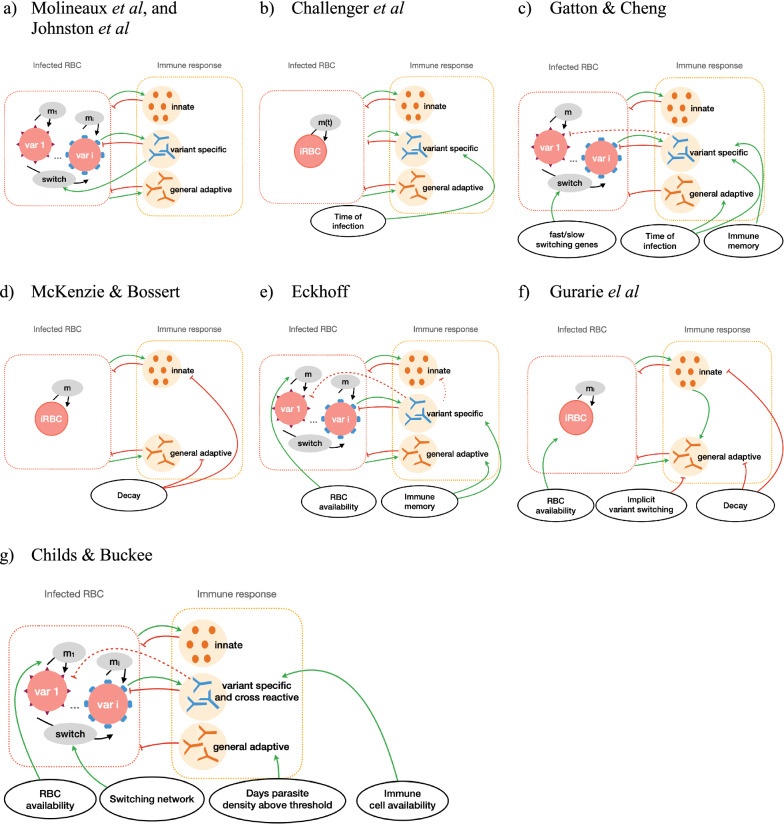
Schematic overview of the main within host dynamics. A simplified representation of the main immune and parasite dynamics for **a** Molineaux et al*.* and Johnston et al., **b** Challenger et al., **c** Gatton et al., **d** Mckenzie and Bossert, **e** Eckhoff, **f** Gurarie et al*.,* and **g** Childs and Buckee. Each model is represented by the main parasite components (left box), the host-immune component (right box) and additional factors influencing parasite dynamics (bottom circles). Models either describe overall infected red blood cells (iRBC) or total iRBC result from a sum of variant specific iRBC (var 1,..,i). Asexual multiplication (m) and variant switching of the parasite are represented by black arrows, and feedbacks between parasite and host components are represented by green arrows or red bar-headed lines for positive or negative effects, respectively. The weaker effect due to cross-reactive immune response is represented by dashed bar-headed lines. A more detailed description of the models can be found in the supplementary material, or in the source publications [17, 18, 21–26]

Each model was reviewed via their detailed descriptions and equations in their respective publications. The models are fully described in the Additional file 1 and their main characteristics are described in Table 1, with more details of their immune response dynamics summarized in Table 2. The type of equations used were classified along with the level of stochasticity integrated into the models, and detailed which dataset/s were used for calibration. Further categorization included the key features of parasite growth and immune dynamics for each model and are summarized in tables (Tables 1 and 2). Equations were classified and divided into four descriptions (1) parasite growth defined by the merozoite multiplication factor; (2a) triggering of, and effect of, the innate immune response; (2b) PfEMP1 variant-specific immunity dynamics; and (2c) the general or non-PfEMP1 immune response dynamic. Figure 2 illustrates the simplified dynamics and feedback between the host and parasite for the different models. The description of the parasite growth in each model is described in Table 1 (row “Assumed multiplication rate”) and the immune responses are described in Table 2. The complete description of each model is given in Additional file 1, and a brief summary is provided here.

**Table 2:** Overview and main immune dynamics of the models

General model structure, and parasite dynamics								
Models	Molineaux et al.			Gatton & Cheng	Eckhoff	Childs & Buckee	Gurarie et al.	McKenzie and Bossert
Adapted models		Johnston et al.						
			Challenger et al.					
Assumptions on innate immune response	Dependent on total parasite density at a given time				Dependent on density of each parasites expressing variants for which there is no antibody response, and on the rupturing of iRBCs	Dependent on total parasite density at a given time and is capped by a maximum efficacy term	Dependent on iRBC density	Dependent on cumulative asexual parasite density
Assumptions on variant specific immune response	Dependent on the variant specific parasite density, and lasting in time with a decaying intensity		Dependent on the total parasite within a time frame preceding the time of response, and on time after infection start	Triggered by a variant specific parasite density threshold, dependent on the time after infection start, and the magnitude increases if the antibody has been produced previously	Dependent on the variant specific parasite density	Dependent on the variant specific parasite density, a maximum growth and decay rate, and is restricted by total number of immune cells available	Not explicit, but adaptive immune response includes random falls due to implicit variant switching	–
Assumptions on general adaptive immune response	Depends on cumulative total parasite density			Dependent on the time after the start of the infection	Modelled as immune response against merozoite antigens, increase every 2 days, dependent on parasite density	Dependent on cumulative number or days the total parasite population is above 10^7^	Dependent on iRBC density and combined innate and adaptive effector pool	Dependent on cumulative asexual parasite density, does not decay in time
Includes cross-reactivity	No		Not explicitly	Yes, the variant specific response approximately kills 5% of all other variants	Yes, response to variants randomly assigned to 5 subgroups	Yes, response to variants randomly assigned to subgroups	No	Yes, immune response to one genotype can be activated by the presence of another genotype
Other comments	Innate and adaptive immune response include patient specific parameters			Parameters differ with different parasite strains	Immune memory is included in the model	Detailed sensitivity analysis resulted in significant impact on the model’s outcome of small changes in parameter values, highlighting the challenged face by within host models		

The model description includes the main characteristics of innate, variant specific, and general adaptive immune responses

### Model specificities

Molineaux et al*.* describe a mechanistic malaria parasite growth model including three components of the immune response, namely an innate immune response acting early in the infection; a variant-specific immune response for each variant-specific parasite population; and a general adaptive immune response acting on total parasite population. This model was intended to reproduce parasite densities from patients in the malariatherapy dataset [12], and describes each of the 50 variant-specific parasite populations within an infection. The model of Molineaux et al*.* was adapted by Johnston et al*.* by replacing two patient-specific parameters which trigger innate and general adaptive immune response with values drawn from a distribution (see Additional file 1). Challenger et al*.* further simplified the model by tracking total parasite density instead of 50 variant-specific parasite densities to reduce memory and computational requirements. The Johnston et al*.* and Challenger et al*.* models are referred to as Molineaux-adapted models in this manuscript.

At a similar time to the publication of Molineaux et al*.*, Paget-McNicol et al*.* [30] published a stochastic model also including three immune response components and exploring different assumptions on switching dynamics of the PfEMP1 variant expression. This model was later adapted by Gatton and Cheng [24]. Instead of a set of discrete model equations with stochastic parameters describing the asexual parasite density in the blood as in Molineaux et al*.*, the Gatton and Cheng model is stochastic and represent the total number of asexual parasites as a decision tree, where parasite numbers trigger choice of path in this decision tree and corresponding model equations [24]. This model differs significantly from Molineaux et al*.* in regard to assumptions around the variant switching dynamics. Additionally, Gatton and Cheng include cross-reactivity between variant-specific immune responses, which was not present in the model of Molineaux et al*.,* although the general adaptive immune response might capture the effect of cross-reactivity implicitly.

More recently, two additional models by Eckhoff [19] and by Childs and Buckee [21] were developed. Eckhoff’s model represents both continuous events such as immune responses to iRBC and discrete events such as the bursting of schizont and the associated immune responses. It also includes immune memory, capturing faster immune responses when an individual is re-exposed to a previously seen parasite variant. Additionally, it includes cross-reactivity between variant-specific antibody responses. In the early infection days, the probability of switching to a new variant increases with growing parasite population. The model developed by Childs and Buckee [21] is deterministic and in their work they explored a wide parameter space to assess variations in the infection dynamics with parameter choice. This model includes four different immune responses, namely innate; variant-specific; general adaptive immune responses; and similarly to the models of Eckhoff, and of Gatton and Cheng, includes cross-reactive immune response across parasite variants. Childs and Buckee investigate different variant switching dynamics, and explicitly limit parasite growth by available RBCs and competition of immune cells for each variant-specific immune response.

Despite these models incorporating detailed variant switching, the malariatherapy calibration datasets contain no information on gene expression profiles. Thus, there is no data describing variant expression and variant switching dynamics in the included patients. Instead, the models utilized understandings from the literature at the time of model development on potential structured switching; some of the models emphasize and discuss the uncertainty around the switching assumptions. Molineaux et al*.* and Johnston et al.assume that immune pressure drives the switch to the expression of a new variant, based on biological studies with *Plasmodium knowlesi* infections in monkeys [7]. In contrast, Paget-McNicol et al*.* refute the theory that switching depends on immunity because it can also be observed in vitro [31], and Gatton and Cheng’s modified model also assumes no link between variant switching mechanisms and host immune pressure. To avoid all variants being expressed with equal chances, the models either assume different switching probabilities for each variant according to a geometric distribution (as in Molineaux et al*.*, and in Johnston et al*.*), limit the number of available variants to switch to in each cycle (as in Eckoff), or assume two groups of variants, with either fast or slow switching behaviour (as in Gatton and Cheng). The model in Childs and Buckee explores different switching networks where all variants can switch to all other variants but with different probabilities and favouring some variants over others (which was adapted from [32]).

The two last models in this review, Gurarie et al*.* and McKenzie and Bossert, are simpler models which do not include variant switching and variant-specific immune responses: thus, including only two immune response components. Gurarie et al. propose a discrete model that assumes the effect of the adaptive immune response is forced to have “random falls” which implicitly allow for the immune response to lose effectivity when the parasite switches to a new variant. These falls in immune response decrease in magnitude during the course of an infection, as it is assumed that the general adaptive immune response builds up. The model of McKenzie and Bossert is the only model presented here which is built around a set of differential equations in a continuous time frame. Capable of representing the general infection pattern (high initial peak followed by a decrease in parasitaemia), the model is not designed to reproduce consecutive peaks and droughts observed in the malariatherapy time-series data. This model was developed to allow for different genotypes to infect the host, and to explore the effect of different gametocyte dynamics on malaria transmission. This model does not include variants nor allow for variant switching dynamics to impact immune dynamics.

### Including inter-individual variability

**Fig. 3:**
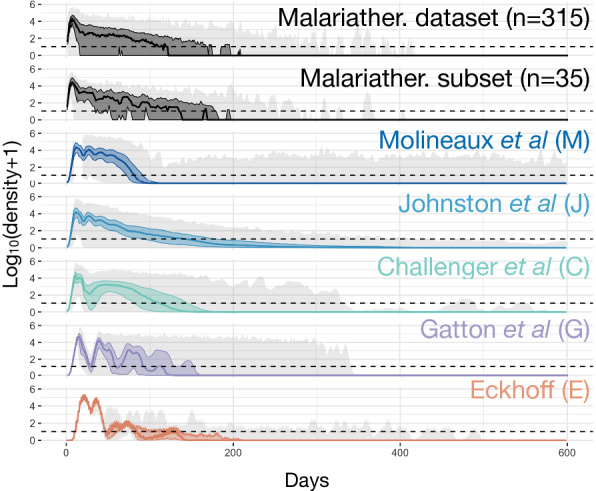
Observed and predicted total asexual parasite density. From top to bottom, the observed log_10_ asexual parasite density in time of the 315 patients from the malariatherapy dataset, the 35 patients from the malariatherapy dataset, and the predicted log_10_ asexual parasite density in time from the Molineaux et al*.* (M), the Johnston et al*.* (J), the Challenger et al*.* (C), the Gatton and Cheng. (G), and the Eckhoff simulations (E). The solid line represents the median across the 1750 simulations per model or patients for the dataset, the colored shaded area the interquartile range (Q25–Q75) and the light grey shaded area the minimum and maximum. The horizontal dashed line indicated the threshold of positive observation (10 PRBC/μl)

**Fig. 4:**
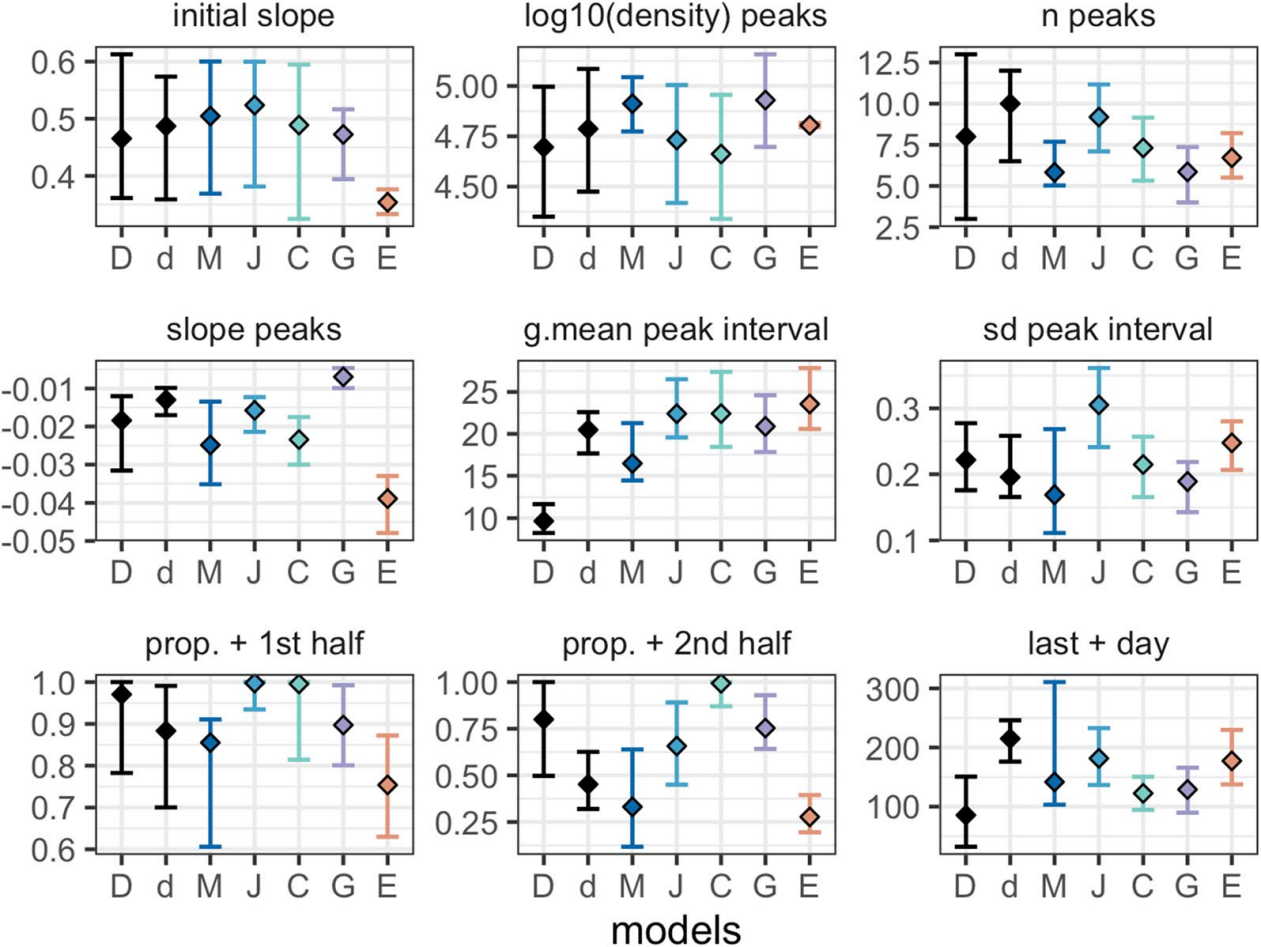
Descriptive summary statistics of the observed and predicted time course of total asexual parasite density. The nine descriptive summary statistics as defined in Molineaux et al*.* (see methods), for the dataset and the 5 models (by colour). Squares indicate the median, and bars the interquartile range. In each plot, from left to right, the estimates are shown for the 315 patients (D) and the 35 patients (d) from the malariatherapy, and for the simulated models of Molineaux et al*.* (M), Johnston et al*.* (J), Challenger et al*.* (C), Gatton and Cheng (G), and Eckhoff (E)

The models attempt to replicate time-series observed in subsets of the malariatherapy data, either with formal or less formal fitting, or at least attempting to reproduce the general pattern observed. The Molineaux et al*.* model was fitted to 35 (out of 318) patients from the malariatherapy data, who were spontaneously cured and received no other treatment. Other models used a larger number of malariatherapy patients (Table 1) to be less restrictive and avoid selection biases. More details on model fitting can be found in Additional file 1: Table S3. There is considerable variation in infection dynamics across the malariatherapy patients, both in magnitude and structure of peaks of parasite numbers, and in length of infections (Figs. 3, 4). This inter-individual variability inherently includes detection and clinical measurement error but is also a result of the stochastic nature of the biological mechanisms involved. These dynamics and observed variability are challenging to replicate. In order to capture the variability, models often implement stochasticity either in the parameters defining parasite multiplication rate and/or in the implementation and effect of the innate and general adaptive immune responses (Molineaux et al*.* and Molineaux-adapted); by creating a stochastic model with most variables defined by Binomial or Normal distributions (Gatton and Cheng, and Eckhoff); or by fitting parameters separately to each patient (Molineaux et al*.*, Mckenzie and Bossert, and Gurarie et al*.*). Similarly, the model developed by Childs and Buckee is deterministic but can include different infection patterns by varying parameter values within the ranges specified in the publication [24].

### Time series of parasite density

Time series of parasite density, summary statistics (described in Methods) of true parasitaemia profiles from 315 and 35 malariatherapy patients [12], alongside time series and summary statistics calculated from 1750 simulated parasitaemia profiles for the five models, are shown in Figs. 3 and 4, and in Additional file 1: Table S1 with distribution of infection length in Additional file 1: Figure S3. Although care was taken with new or adapted code, there may be small differences from the original publications.

The observed log_10_ parasite densities of the patients from the malariatherapy data varies significantly across the patients, a variability which appears to be accounted for in the models (Fig. 3). The 35 patient subset included only infections that ended naturally, and excluded acute very short infections. In Molineaux et al*.,* most infections cease before day 200, with a subset of simulated parasitaemia appearing to be chronic infections that do not end before the end of the simulation. The model of Johnston et al*.* increased the variant-specific immune response decay compared to Molineaux et al*.*, thereby decreasing the immune response’s efficiency. This results in increased simulated infection lengths compared to the simulations in Molineaux et al*.* The general shape of decay in parasite densities from Johnston et al*.* also appears to be more exponential-like compared to the other models, which is most likely a result of the stronger effect of the general adaptive immune response. In Challenger et al*.,* simulations indicate a general pattern of two peaks, the first is followed by a decrease as a result of the innate immune responses, followed by a second wave of parasitaemia that is controlled by the variant-specific and general immune responses. Simulations from Gatton and Cheng indicate a slightly different time course of peaks compared to those predicted from Molineaux et al*.* and the models adapted from Molineaux et al. Simulations from Gatton and Cheng indicate a general first peak with a sharp decrease in parasite densities with initial innate immune responses, followed by new peaks in parasite densities given the probabilistic approach taken in Gatton and Cheng (see Additional file 1: Table S2 for the equations)*.* The general decline in predicted parasite densities (decreasing peaks) is not reflected in the summary statistics (Fig. 4). In Eckhoff’s model the first peak is followed by much lower peaks, resulting in a steeper slope of peaks in the summary statistics (Fig. 4). The average infection lengths are consistent with the 35 malariatherapy dataset, although it is worth noting that the Eckhoff model was fitted to a larger dataset from the malariatherapy patients including more than the 35 patients in Molineaux et al.

### Total parasite multiplication rate during infection

**Fig. 5:**
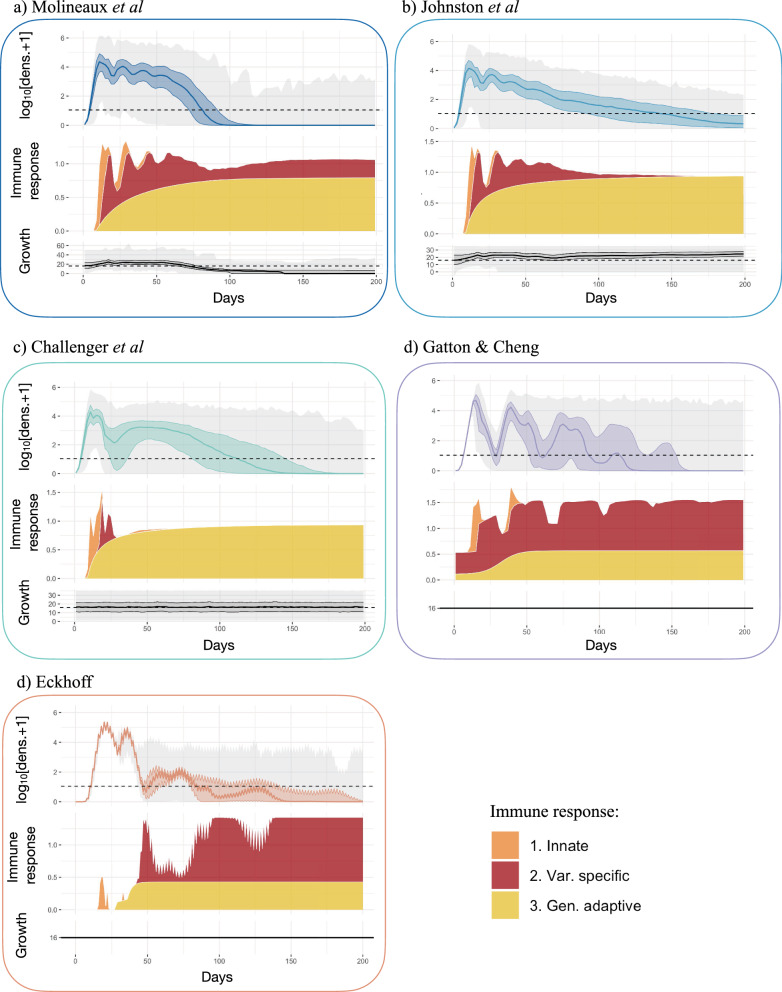
Asexual parasite densities, immune responses, and multiplication rates in different models. Each panel shows, from top to bottom, 1. The log_10_ asexual density as in Fig. 3, with the horizontal dashed black line indicates the threshold of positive density (10 PBRC/μl); 2. The stacked magnitude (as the $|{\mathit{log}}_{2}\left({R}_{t}+1\right)|$ with ${R}_{t}$ the median effect of each immune response) of the innate (orange), variant specific (red), and general adaptive (yellow) immune response, such that the maximum effect equals 1 and no effect equals 0; and 3. The overall (across variants, see Methods) multiplication rate, with the horizontal dashed line representing a multiplication rate of 16. The x-axis represents days of infection. Results are shown for **a** Molineaux et al., **b** Johnston et al., **c** Challenger et al*.,* **d** Gatton and Cheng, and **e** Eckhoff. The x-axis represents days of infection, the solid line the median, the shaded area the interquartile range (Q25–Q75) and the grey lines the minimum and maximum resulting from the 1750 simulations

Each variant-specific parasite density in the simulations was tracked in all models except Challenger et al*.* and Eckhoff, the former because the equations do not explicitly model each variant and the latter due to the computational complexities of tracking each variant. The average multiplication rate at each time step is shown in Fig. 5. For Gatton and Cheng, and for Eckhoff*,* the multiplication rate of the parasites, defined as the multiplication rate without the effect of any host immune response, is fixed at 16 per cycle and remains the same for all simulations and time (Fig. 4d, e). For Johnston et al*.* and Molineaux et al*.,* each variant-specific parasite has its own multiplication rate drawn from a Normal distribution with mean of 16. For each time step, the overall multiplication rate for Johnston et al*.* and Molineaux et al*.* is thus the weighted average of the variant-specific multiplication (see Methods).

Therefore, these two models have a constant multiplication rate, in time, for each variant-specific parasite. However, the overall multiplication rate fluctuates over time, representing the competition between variant-specific parasites (with different multiplication factors), with new variants appearing, and other variants disappearing throughout the infection. For some simulations (Molineaux et al*.* and Johnston et al*.*), the overall multiplication rate is high at the beginning of the infection (Fig. 5a, b) and causes a peak in parasitaemia density. The predicted infection length for a subset of simulations from both Molineaux et al*.* and Johnston et al*.* is very long, and in these subsets of simulations, the overall multiplication rate is in fact high throughout the infection (see Additional file 1: Fig. S2).

**Fig. 6:**
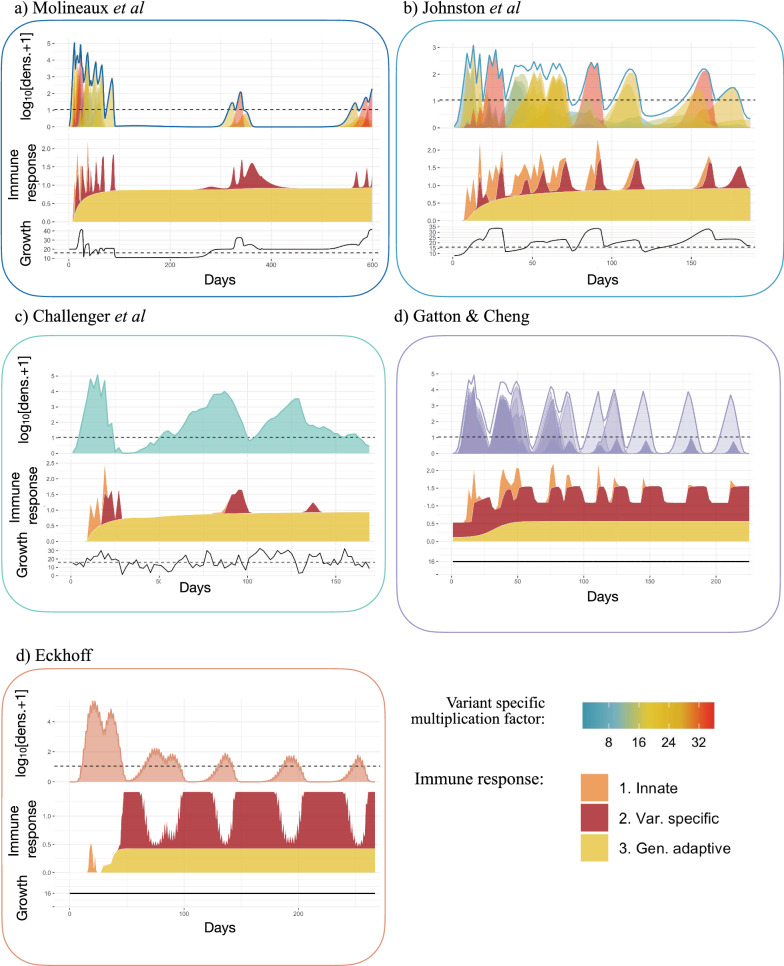
Example of asexual parasite densities, immune responses, and multiplication rates in single simulations from different models. Each panel shows, from top to bottom, 1. the log_10_ asexual density and variant specific asexual parasite density, with the horizontal dashed black line indicates the threshold of positive density (10 PBRC/μl); 2. The stacked magnitude (as the $|{\mathrm{log}}_{2}\left({\mathrm{R}}_{\mathrm{t}}+1\right)|$ with ${\mathrm{R}}_{\mathrm{t}}$ the effect of each immune response) of the innate (orange), variant specific (red), and general adaptive (yellow) immune responses, with 1 representing maximum effect and 0 no effect for each immune response type; and 3. The overall (across variants, see Methods) multiplication rate, with the horizontal dashed line representing a multiplication rate of 16. The x-axis represents days of infection. Results are shown for **a** Molineaux et al., **b**. Johnston et al., **c** Challenger et al., **d** Gatton and Cheng, and **e** Eckhoff

In Challenger et al*.*, the multiplication rate at each time step is an input parameter of the model. It is both drawn from a Normal distribution with mean 16 and correlates with the previous time step [25] (see Additional file 1: Table S2). Thus, the median multiplication rate across all simulations remains constant at 16 by design (Fig. 5c). Taking the example of a single simulation (Fig. 6), the pattern of overall parasitaemia in the Challenger model is less driven by the average growth rate in time compared with the simulations for Molineaux et al*.* and Johnston et al*.* (Fig. 6a–c). Molineaux et al*.* reported that a varying overall parasite multiplication rate, and thus the parasite inherent proliferation rate (without immunity), is essential to recreate observed peaks in parasitaemia in the dataset. Furthermore, long-lasting infections only occurred in models that express highly multiplying variants. Overall, this indicates infection dynamics in Molineaux et al*.* and Johnston et al*.* are primarily driven by stochasticity in the inherent growth of the parasites, whereas in Challenger et al*.,* Gatton and Cheng*,* and Eckhoff, the stochasticity in their predicted dynamics is driven more by immune responses and subsequent killing effects.

### Immune response dynamics in the models

In terms of immune response variability between the simulations (and thus assumed to exist between individuals), the highest variance across individuals arises from the variant-specific immune response, followed by the innate immune response, with the general adaptive response being reasonably consistent across individuals for the models (Additional file 1: Fig. S3). The key assumptions around immune dynamics for each model are summarized in Table 2. The simulated immune responses are shown for five models in the middle row plots in Fig. 5, and examples of single simulations are shown in Fig. 6. Note that in Gatton and Cheng and in Eckhoff, the effects of the immune responses are drawn from Binomial distributions; thus, the variability in the effects of the immune responses are not entirely captured in the immune time plots of Figs. 5 and 6.

The innate immune response is mainly active at the beginning of the infection (approximately the first 20–50 days in the different models) as it responds directly to high levels of parasite density. In Eckhoff’s model, the innate immune response is modulated by the adaptive immune response, reducing with increasing antibodies against the parasite population, making this model the only one with direct feedback between innate and adaptive immune response.

The variant specific immune response, effective against variant-specific parasite after delay, is modeled for each variant explicitly in Molineaux et al*.*, Johnston et al*.*, Gatton and Cheng, and Eckhoff. The increased decay rate in Johnston et al*.* compared to Molineaux et al*.* leads to a reduction in the immune responses (Fig. 5). In Challenger et al*.*, once the variant-specific immune response is activated, it is quickly efficient in reducing parasite densities compared to the other two models (steeper slope in the variant-specific immune response curve in Fig. 5 and Additional file 1: Fig. S2). This efficient immune response likely explains the steep decrease after the first wave of parasite density. In Gatton and Cheng, the variant-specific response is activated when the variant-specific parasite density reaches the threshold of 12 PRBC/μl [24], and the magnitude is increased if the antibody was already produced during the infection. When there are high peaks throughout the infection, the variant-specific immune response remains very active throughout the predicted infections from Gatton and Cheng*.*

The general adaptive immune response increases in time since the start of the infection, responding to the cumulative parasite density (Molineaux et al*.,* Molineaux*-*adapted*,* Eckhoff, McKenzie and Bossert, and Gurarie et al*.* models), time (Gatton and Cheng) or a combination of both time and parasite density (Childs and Buckee). An important finding is that by comparing all three immune responses and the total net immune response (Figs. 5 and 6), the general immune response is the main effector of reducing parasite density in all models approximately after 50 days from start of infection. However, the variant immune response will ultimately end the infection. The decrease of the critical density for the general adaptive immune response in Johnston et al*.* and Challenger et al*.,* compared to Molineaux et al*.* (see model description and Additional file 1: Table S2) leads to a higher effect of the general adaptive immune response in those models, which compensates for the weaker effect of the variant-specific immune response.

## Discussion

In this study, eight published mechanistic within-host models of the asexual blood-stage dynamics of *P. falciparum* were reviewed, five of which were reproduced via simulation analysis. Several features and simulation outputs from the models were compared including the predicted time-series of asexual parasitaemia, modelled growth rates, innate immune responses, variant-specific immune responses, and general adaptive immune responses. The models varied widely in complexity. Rather simple models such as McKenzie and Bossert have the advantage that they do not rely heavily on assumptions of unknown biological mechanisms, while more complex models, such as Eckhoff or Childs and Buckee capture more detailed, yet less well understood, immune and parasite mechanisms. Understanding the variation in multiplication rates, versus immune and other host factors, or random effects and measurement error, and their impact on parasite density variations is particularly important when the models are included in broader investigations of the effect of a vaccine, drug or other interventions aimed to modify parasite growth patterns. The overview presented here provides a general understanding of those models.

### Model composition varies in complexity and uncertainty

Parasite and host dynamics are represented in the mechanistic models via detailed description of the parasite replication dynamics, and up to four host immune responses. Each model has its own additional complexity, specifications, and advantages. For example, to obtain increased detail of the host’s response, some models include red blood cell availability and limit the maximum immune response capacity. Or, to include more details in the parasite dynamics, some models include specific variant switching mechanisms. Models generally define a negative feedback loop between the parasite density or cumulative parasite density since the start of the infection and the effect of the immune responses, and in addition some models add the time of infection (Gatton and Cheng*,* Childs and Buckee, Eckhoff) as a determinant for the magnitude of the general immune response.

Including stochasticity in the models is particularly relevant for the within host dynamics of *P. falciparum* as the infection patterns observed in the malariatherapy data are highly variable among patients. Yet this is a challenge for modellers as it requires more complex models that include stochasticity, and it requires capturing inter-individual variation in the fitting process. Molineaux et al*.* proposed nine summary statistics for the 35 malariatherapy patients as outcomes of interest to be reproduced. Other models have used those summary statistics to describe the dataset, although most did not include all nine. Given the limitations of the malariatherapy dataset, the models should not aim to reproduce all nine features, nevertheless characteristics such as a wide range of infection lengths, a high early peak representing the acute infection phase, sometimes followed by chronic infection, should be accounted for. In the context of within host models used for modelling transmission and impact of interventions, additional key features to capture, which were not included in the current analysis, are infectiousness and symptomatology.

Our analysis highlighted that the Molineaux et al*.* and the Molineaux-adapted models have likely allocated too much stochasticity to the individual parasite multiplication rates, thus masking other mechanisms, and placing relatively less importance on immune responses and other host factors. Furthermore, for these models, their assumptions concerning the inherent multiplication rates of the parasites differ from other models, along with assumptions of large variability in the variant-specific parasite multiplication rates in the absence of any immune response. These dynamics were found to be essential in the Molineaux et al*.* and Molineaux-adapted models to reproduce clinical malariatherapy patterns of infection, rather than immune responses [22]. In particular, in Molineaux et al*.* and Johnston et al*.,* longer infections result from the expression of variants with a high multiplication rate towards the end of the infections for later variants. In contrast, the other models did not rely on variation to capture infection patterns. Instead, variation was mainly included in the control of infection due to immune responses and switching mechanisms.

For models including multiple parasite variants, the variant switching dynamics are an important mechanism driving the parasitaemia predictions. The switching dynamics define how the parasite goes from expressing one PfEMP1 variant to another one at the next generation to evade the immune response. Switching dynamics in the models have been assumed to respond to the variant specific immune response (Molineaux et al*.* and Johnston et al*.*), to the current variant population size (Eckhoff), or were determined by more sophisticated switching networks (Childs and Buckee, Gatton and Cheng). Both the variant switching dynamics and the variant-specific immune response are essential drivers of infection patterns and inter-individual variability in all models (except McKenzie which does not include variants). With detailed *var* gene transcription analysis studies limited to early days of infection in volunteer infection studies (VIS) [33–35], data available to inform the models on switching dynamics over the entire course of an infection remain insufficient.

The various assumptions around the mechanisms of action of the different immune responses and the interplay between parasite and host highlight the challenge of realistically reproducing the time-series observed in the malariatherapy dataset, especially the inter-individual variability. This challenge is confounded since there is limited knowledge of the biological mechanisms at play.

### Parasite multiplication rates might be lower than initially assumed

It is commonly agreed that a single iRBC produces 16 merozoites [3], of which a portion successfully invade new erythrocytes. Growth rates in vivo are more difficult to measure, and compared to the assumed multiplication factor of 16, includes the host-parasite interactions, which reduce the observed parasite growth. Several independent statistical models previously estimated parasite growth at onset of infections in both malariatherapy or VIS. Estimates from the malariatherapy dataset range between 10 and 18 [36], or 6 and 24 [20]. In malaria VIS the growth factor was estimated to be between 12 and 15 [37], and in the control cohort of vaccines AMA1-based vaccine challenge between 14 and 21 [38]. More recently, estimated ex vivo multiplication factors for different malaria genotypes were found to be between 2–11 for laboratory strains and new clinical isolates [39]. One hypothesis explaining the variation among parasite growth relies on the differential capacity of the PfEMP1-variants to evade splenic clearance, with the hypothesis that a subgroup of PfEMP1 expressing parasites might be fast growing (due to increased cytoadherence and thus decreased splenic clearance) [40]. This mechanism may explain differing growth rates among parasites expressing different variants, and explain apparent higher multiplication rates in naïve individuals if their parasites express the fast growing PfEMP1 subgroup [40]. Variance in parasite multiplication rates among clones and among infections across individuals is possible due to this variance in successful avoidance of splenic clearance during blood-stage replication, however, it is unclear whether the range should include an overall multiplication factor as large as 32 or 35, as in some models reviewed here (namely in [21–23, 25]). RBC availability might also constrain successful invasion of RBCs and thus affect the effective replication of the parasite. Recent in vitro studies highlighted distinct RBC invasion strategies of *P. falciparum* strains, with parasites that favour RBCs of different age [41], and different parasite strains either invading a larger fraction of RBCs at lower rates or invading smaller fraction of RBCs at a higher rates [41]. In addition to potential age-dependent differences in RBC availability, it is known that certain RBC polymorphisms, for example sickle cell traits and blood groups [42] impact the invasion of RBCs. These studies suggest that variability in effective parasite growth, both within host and across individuals, might be attributable to heterogeneous RBC accessibility and susceptibility to parasite invasion.

### Variant switching and immune response modeling are limited by current knowledge

Switching mechanisms are not well understood, and it remains unclear if the switching mechanisms are driven by antibody response (as in [22]) or are not directly influenced by the immune pressure (as in [21, 24]). In contrast to the assumptions made in the models reviewed here, it is likely that parasites express more than one variant, if not all variants, during the first blood stage generation [34]. As highlighted by Childs and Buckee [21], this finding challenges the current understanding on the underlying mechanisms leading to chronic infections. Cross-reactivity, suggested as a mechanism necessary for chronic infections [43] and included explicitly in Gatton and Cheng, Childs and Buckee, and in Eckhoff would not allow for chronic infections if all variants are expressed at infection onset [44], and models would have difficulties to recover long infection patterns. The lack of understanding about the switching dynamics supports the assumptions in Challenger et al*.*, as they only model total parasitaemia without modeling switching between variants. The models described by McKenzie and Bossert, and Gurarie et al*.*, are less complex and do not model variant-specific parasitaemia, offering potential modeling alternatives when detailed mechanisms of immune response are not needed. To our knowledge, there are few biological studies on the kinetics and interplay of the immune responses as defined by the models (innate, variant specific, and/or adaptive immune response). As such it is unclear how much each immune component affects the overall time course of infection. Therefore, it is not surprising that models differ in the relative importance of general or variant-specific immune responses. Moreover, in the absence of a clear biological understanding of the variability in infection time-series, model stochasticity remains an important driver of the modelled immune and parasite dynamics.

### Further data for new models

Blood samples can only inform on the level of circulating parasites (and associated measures), thus current tools are blind to parasites while sequestered and hidden in capillaries to avoid splenic clearance [1]. Consequently, it remains difficult in humans to experimentally assess localized host-parasite interactions for a full understanding of the role of different immune actors and potential resource limitation. Nevertheless, a few data sources on circulating parasites are available.

It is essential to highlight here that the malariatherapy dataset cannot be considered as a typical time course of an infection, as the data come from patients with severe neurosyphilis, who were malaria naïve (although previous infection cannot be completely ruled out as some patient lived in area where malaria was endemic at that time), and did not include children. Furthermore, inoculations were limited to a restricted set of parasite strains, measurements were prone to errors, and data did not include any measurements which could directly inform immune response and RBC dynamics, nor parasite gene expression dynamics. Thus, although the malariatherapy data are the only detailed data available on infection time-course, models are not strictly evaluated by their ability to reproduce malariatherapy-like infections. Data on early infections are available in high detail from VIS. They provide precise quantification of parasitaemia at much lower detection threshold than the malariatherapy data, and variant expression dynamics or other genetic traits relevant for understanding the dynamics in the first few days of blood stage infection can now be informed by such studies. Note that most of the parasite densities measured in VIS fall below the 10 iRBC/μl detection threshold in the malariatherapy records, and untreated infections last ten days at most. Thus, most parasitaemia levels available from VIS would probably correspond to pre-recorded infection times in the malariatherapy records, making a direct comparison between the two datasets difficult. Beyond early infections, models need to rely on longitudinal field data to assess their performance. Longitudinal field data are extremely important to explore the dynamics in realistic settings, with individuals living in endemic areas who are repeatedly exposed to malaria, including children who are most at risk for the disease, and including a range of genetic diversity and complexity of infection. Because immunity builds up with age and exposure [45], and genetic diversity is a result of immune pressure, longitudinal and cross sectional field studies which include genetic analysis give important insights in malaria infection dynamics.

The current analysis and review focused on infections in naïve individuals and did not include a review of the models for their ability to capture infections in pre-exposed individuals. Although data is lacking, the immune effect of pre-exposure could be added to the models as a second step, for example by adding an overall reduction factor that would lower the magnitude of the parasite density in function of age and/or exposure, similar to an empirical model by Maire et al*.* [46]. The effect of co-infection was not included here and its implementation was a focus of the Childs and Buckee’s model [21], which hypothesizes that co-infections and super-infections have different effects based on the timing of the second infection, and that the effects of multiple infections seem to be poorly understood, and thus poorly included in models [21]. Recently many field studies focusing of genetic data and analysis are giving insights in the effect and dynamics of multiple infections on a population scale (for example [47, 48]), yet empirical data on the time course of complex infections are sparse and insufficient to validate models of co-infections, relying on data from mouse models for detailed infection dynamics [49, 50].

## Conclusions

This review provides insights on existing models of asexual *P. falciparum* blood-stage infections, and some insights on both known and unknown biological mechanisms driving infection dynamics. Blood-stage parasite densities are at the core of malaria transmission, morbidity and mortality. Thus, population models that include models of within-host parasite dynamics to estimate the impact of blood-stage drugs or vaccine, or estimate the impact of parasite resistance, should be aware of the underlying assumptions made in the within-host model and how those changes effect infection dynamics. Complex within host models offer a great range of hypothesis on unknown parasite and host mechanisms, which is an end in itself, but in the context of implementing within-host models in broader transmission models, simpler models might be equally useful.

## Supplementary Information

**Additional file 1.** Summary description of each model. **Figure S1.** Schematic illustration of an infection and variant specific immune responses at a given time point from models Molineaux et al., Johnston et al., and Challenger et al..**Table S1.** Summary statistics of the observed malariatherapy data and the models. **Figure S2.** Immune responses, multiplication rates and asexual parasite densities in different models. **Figure S3.** Distribution of infection length in the malariatherapy dataset and in the models. **Table S2.** Model equations

## Data Availability

The outputs generated and analysed during the current study are available from the corresponding author on reasonable request.

## Abbreviations

IBM: Individual-based model
(i)RBC: (Infected) red blood cell
PfEMP1: *Plasmodium falciparum* Erythrocyte membrane protein 1
*P. falciparum*: Plasmodium falciparum
VIS: Volunteer infection studies
