# Supplementary material for: Mechanistic within-host models of the asexual Plasmodium falciparum infection: a review and analytical assessment
Source: Malar J. 2021 Jul 10;20:309. doi: 10.1186/s12936-021-03813-z (PMC8272282; doi:10.1186/s12936-021-03813-z)
Supplement: Supplementary file 1 — Additional file 1. Summary description of each model. Figure S1. Schematic illustration of an infection and variant specific immune responses at a given time point from models Molineaux et al., Johnston et al., and Challenger et al..Table S1. Summary statistics of the observed malariatherapy data and the models. Figure S2. Immune responses, multiplication rates and asexual parasite densities in different models. Figure S3. Distribution of infection length in the malariatherapy dataset and in the models. Table S2. Model equations [file 12936_2021_3813_MOESM1_ESM.pdf]

Additional file

Mechanistic within-host models of the asexual *Plasmodium falciparum* infection: a review and analytical assessment

Authors:

Flavia Camponovo[1,2,3], Tamsin E. Lee[1,2], Jonathan R. Russell[4], Lydia Burgert[1,2], Jaline Gerardin[5], Melissa A. Penny[1,2*]

[1] Swiss Tropical and Public Health Institute, Basel, Switzerland

[2] University of Basel, Basel, Switzerland

[3] current address: Center for Communicable Disease Dynamics, Department of Epidemiology, Harvard T. H. Chan School of Public Health, Boston, MA 02115, USA

[4] Institute of Disease Modeling, Bill & Melinda Gates Foundation, 500 5th Ave N, Seattle, WA 98109, USA

[5] Department of Preventive Medicine and Institute for Global Health, Northwestern University, Chicago IL, USA

[*] corresponding author

Summary of each model

Molineaux *et al. (1)*: The model was developed to reproduce spontaneously cured malaria infection observed in 35 patients of the malariatherapy dataset who were not treated throughout the course of the therapy. The primary aim of the model was to incorporate realistic host immune responses and parasite dynamics, and to eventually include the model in a population transmission model at a later date.

The model described by Molineaux *et al.* is the first model in which immune responses against infection were divided into three components, *i)* the innate immune response, *ii)* the variant specific immune response and *iii)* the general adaptive immune response (variant transcending) (for details see Table S2). It is a discrete model with a 2-day time step to align with the merozoite's 48 hour replication cycle, and the model allows for up to 50 variants of the parasite population. At each time step the observed parasite growth increases as a result of the intrinsic parasite multiplication rate and is reduced by the effect of the three immune responses (with each response ranging between 1 = no effect to 0 = maximum effect). Each infection begins with all 50 variants (denoted by i = 1,2,…,50) with initial densities set at 0, except one variant set at 0.1 PBRC/μl. At each time step parasites switch to different variants, with variant specific switching probabilities defined by a constant switching rate. The different probability of switching to each of the variants follows a geometric distribution and is modulated by the variant specific immune responses.

The innate immune response is thought to be triggered early in the infection (first few weeks), and its effect is proportional to the total parasite density at a given time, conditional on a host specific critical density. The variant specific immune response is triggered by the variant specific parasite density, effective after a certain delay (8 days), and persists with a decaying intensity. In addition, the switching mechanisms are controlled by the variant immune response, with the probability to switch to given variant increasing with high total immune responses against each of the 50 variants and decreasing with high immune response against that specific variant. The variant specific immune response, and the capacity of the parasite to switch to new variants, are thought to be a major cause for the different waves of parasitemia, and the chronic pattern of infection. The general adaptive immune response builds up slowly against the conserved part of the parasite, and acts against all variants of the parasite equally. It is effective after a delay (8 days), conditional on a host specific critical

density, and responds to the cumulative parasite density with no decay in time. The general adaptive immune response is assumed to be the main cause of infection clearance, and this is confirmed by simulations except for a few simulations with chronic infection a result of stochasticity in several variant growth rates (see Fig 4 in the main).

In this discrete model, the authors include both patient specific and stochastic parameters to represent the high variability in infection dynamics among the 35 malariatherapy patients (see parameters P_m and P_c, in Table S1). Variant specific parasite multiplication rates are drawn from a Normal distribution with a mean of 16 (truncated at a minimum of 1), leading to different multiplication rates for each of the 50 variants and for each simulation, but constant throughout the infection and different in each simulation. The inter-individual variability is captured by two patient specific parameters representing the critical densities for the innate immune response and the general adaptive immune response. The critical densities are calculated using the first local maximum density, and the length of the infection directly observed for each patient in the dataset.

Molineaux *et al.* additionally added a measurement error and thus were able to reproduce the 35 infections with their model by selecting, as a final step and not included in this analysis, one simulation from a pool of 50 simulations, a pool generated after trial and error, that best fit the min, median, max of the nine summary statistics, using χ^2 test, for each for the 35 patients. This final step makes it difficult to reproduce the authors selection, but general infection dynamics for each patient are possible to reproduce.

Johnston *et al. (2)*: The model was directly adapted from Molineaux *et al.* in order to be included in an individual-based model (IBM), with four parameters re-fitted via bootstrapping method to fit the minmedian and max of the nine summary statistics as well as duration o infection (more details in Table S3). This transmission model was used to understand the human component of the reproductive number R_0, and to investigate the impact of drug treatment (through Pharmacokinetic and Pharmacodynamic (PKPD) models) and drug resistance on transmission (2). To include an adapted Molineaux *et al.* model in a transmission model, they modified the within host model to avoid the two patient specific parameters. Instead, these parameters were drawn from appropriate distributions to maintain inter-individual variability. The authors re-parametrized the patient specific parameters by using the log Normal distribution (for estimates of the first local maximum density) and the Gompertz distribution (for estimates of the length of infection) defined in previously

published literature (3). To maintain the parasite multiplication rates in Johnston *et al.*within a smaller range, and thus a biologically more realistic range compared to Molineaux *et al.*, the Normal distribution was truncated not only at 1, but also on the upper end at 35.

In order to obtain a reasonable fit to the dataset following modification to the critical densities for the innate immune response and the general adaptive immune response, Johnston *et al.* re-fitted three additional parameters. All other parameters were fixed to values from Molineaux *et al.*. The refitted parameters are, *i)* the decay parameter of the acquired variant specific immune response (σ) was increased from 0.02 to 0.15, and *ii)* a constant (k_m) allowing the calculation of the critical density threshold used for the general adaptive immune response (P_m) was reduced from 0.04 to 0.025, and *iii)* a constant (k_c) allowing the calculation of the critical density threshold used for the innate immune response (P_c) was reduced from 0.2 to 0.164. By increasing the decay rate of the acquired variant specific immune response by almost ten-fold, Johnston *et al.* effectively increased infection length as an increased variant specific immunity decay rate (compared to Molineaux *et al.*) leads to a less efficient variant specific immune response. In contrast, parameters k_m and k_c in Johnston *et al.* are coupled to P_m and P_c and potentially increase the strength of the innate and general immune response (see Table S2 for full details).

Challenger *et al.* *(4)*: Challenger *et al.* adapted the model from Johnston *et al.*, with the aim to assess the impact of treatment adherence and treatment failure (adding PKPD). The main adaptation of Johnston *et al.*, by Challenger *et al.* was reduction of the 50 variant specific parasite densities to a single total parasitemia, thereby reducing the number of equations from 50 to 1, simplifying the model and reducing computational time and memory requirements. The variant specific immune response in Challenger *et al.* is thus a function that represents the response to all variants instead of being the sum of responses to each variant. Early in the infection, the variant specific immune response is modeled similarly to the innate immune responses of Johnston *et al.* and Molineaux *et al.*, except for a different critical density threshold (constant for the innate immune response, stochastic for the variant specific immune response, see Table S2). During the time course of the infection, the variant specific immunity in this model responds not only to the parasite density eight days before (due to assumed delay in activation), but also to earlier time points. Thus Challenger *et al.,* in contrast to Molineaux *et al.* and Johnston *et al.*, implicitly assume immunological memory to

previously expressed variants and cross-reactivity *(4)*, despite not tracking all 50 variants (see Fig. S1 and Table S2).

Since Challenger *et al.* do not track 50 variants, unlike Molineaux *et al.* and Johnston *et al.*, the model considers an overall multiplication rate across all variants expressed at each time. This overall multiplication rate is computed for each time step, drawn from a Normal distribution around 16 while being positively correlated to the previous time step, implying that this stochastic, but correlated, parasite multiplication rate is the result of the emergence and disappearance of variants during the time course of an infection (4).

The model of Challenger *et al.* was fitted to the same 35 malariatherapy patients as in Molineaux *et al.,* fitting the parameters of the overall variant specific immune response and the overall multiplication rate, and re-fitting the constant allowing the calculation of the critical density threshold for the general adaptive immunity (k_m). The authors used Markov chain Monte Carlo method u(Metropolis-Hasting algorithm) to generate random walks in parameter space and fitted the model to the nine summary statistics (see Table S3). A constant that allowed the calculation of the critical density threshold for the general adaptive immunity (k_m) was reduced from 0.0250 to 0.021 which leads to a slightly stronger general adaptive immune response in Challenger *et al.* compared to Molineaux *et al.*. Additionally, the critical density threshold of the innate immune response function (P_c) was drawn from a log Normal distribution with $\sigma = 1.2$ instead of $\sigma = 1.148$ as in the other models. All other parameters are identical to the model in Johnston *et al.*.

Gatton & Cheng (5)**:** Gatton & Cheng (5) is a model adapted from a previously published probabilistic model of Paget-McNicol *et al.* (6). The model was fitted to the malariatherapy data differentiated by the *P. falciparum* I strain used. The model was fitted using sets of 100 simulations, altering 6 parameters in the model. They compared the model output to four variables, namely maximum parasitemia, number of days with fever, number of days with parasitemia higher than 10/microliter, and number of days with parasitemia higher than 10000/microliter (see Table S3).

As with Molineaux *et al.*, it tracks 50 different variant specific parasites, starts the infection with only one variant expressed, tracks the parasites in 2-day time steps, and includes three different types of immune responses. The model describes the total number of asexual parasites via a flowchart which acts more like a decision tree with triggers different equations

for given parasite numbers (5) (see equations in the Table S2). At each time step the parasites undergo replication with a multiplication rate of 16, and the probability of success of the replication is computed from Binomial distribution dependent on the general adaptive and variant specific immune response. Thus, in the absence of any immune response, the parasites have a constant growth rate of 16.

The innate immune response in Gatton & Cheng is activated after reaching a threshold and is assumed to increase with a higher number of parasites until a maximum effect is reached. The effect of the variant specific immune response is modeled as being inversely proportional to the number of antibodies produced, the latter being computed at each time step. The magnitude of the antibody response is dependent on whether this antibody has previously been produced during the infection, as well as cross-reactivity (so that an antibody response against a specific variant also has a small effect on all other variants). The general adapted immune response is only dependent on the time of infection, thus not changing with parasite numbers, nor does it differ between individuals (see Table S2). Additional biological differences between the model of Gatton *et al.* compared to Molineaux *et al.* and Johnston *et al.* is the switching mechanism from one variant to another, which in Molineaux *et al.* and Johnston *et al.* is assumed to depend on the variant specific immune response, whereas in Gatton & Cheng it is completely independent from the host's immune response. In addition, Gatton & Cheng assumes two populations of variants, either fast or slow switching, with 10 variants considered fast switching (rate ~ 0.5-4.5%) and 40 in the slow switching group (rate ~ 9.10-11.8 %) , compared to 0.02% in Molineaux *et al.* and Johnston *et al.*. Switching dynamics were previously investigated by the author (7) and based on *ex-vivo* analysis of var gene transcripts of parasites isolated two volunteers who underwent challenge infection (8).

Eckhoff (9): A summary and illustration of this model can be found on (10). This model has both discrete and continuous components, to represent discrete events such as schizont rupture in a two-day interval and the antibody response against merozoites, and to represent the continuous immune components acting on infected red blood cells (iRBCs). This model is more complex in the immune response mechanisms described, compared to the Molineaux *et al.* and Molineaux adapted models, as it explicitly includes antibody responses to PfEMP1 and with smaller effect to minor epitopes, it includes immunological memory of the antibody response, and it includes continuous and discrete immune responses.

The blood-stage immune response is divided into four components, namely the innate immune response, the antibody immune response against each PfEMP-1 variant, and each shared minor epitope, and the antibody immune response against merozoite proteins (such as MSP-1 and AMA-1). As for the Molineaux *et al.* and Molineaux adapted models, the innate immune response is assumed to limit the maximum parasite density (first peak). This innate immune response acts against both iRBCs (continuous component) and against ruptured schizonts (discrete component). Its effect is activated by a pyrogenic threshold, and it decreases with increased antibody levels. The antibody response is dependent on the capacity to generate specific antibodies, which depends on the concentration of the variant specific parasite and on the immune memory to the parasite. Similarly, to other models, the antibody response decays once the corresponding parasite population is absent, but the antibody production capacity keeps an immunological memory. Thus, once exposed again to the same variant, the antibody response will reach higher levels faster. The immune response against merozoite proteins has a similar effect as the general adaptive immune response in the other models. It increases in time and leads to a decrease of the parasite peaks amplitude in time. The PfEMP1 variant specific immune response eventually clears the infection.

Initially, the model was fitted to malariatherapy data, but contrary to Molineaux *et al.,* the dataset includes patients with reinfection (as memory of immune response is modeled) and the perturbed ones to avoid selection biases. Parameters are either assumed through literature, or fitted to the malariatherapy dataset (details in (9)). The model outputs were compared to the first parasite density peak, lower secondary peaks, and lower peaks after 100 days, the possibility of reinfection with homologous strains, the interval between peaks, and the distribution of measured durations (9). The model has been re-fitted more recently using field data (11), and re-calibrated parameters included the number of PfEMP1 variants the switching rate, the number of MSP variants in the overall parasite population, the fraction of merozoites inhibited by maximum MSP1-specific antibody level, the number of minor epitope variants, and the kill rate of infected red blood cells due to antibody response to minor epitopes. As described in (11), a Dirichlet-multinomial distribution was used to compare simulation data with field data (12,13).

Childs & Buckee (14): This is a deterministic discrete model with 2-day time steps. It tracks parasite population of 60 variants and includes four different immune mechanisms. The immune response includes innate, variant specific, general adaptive immune response, similar

to the other models, as well as a cross-reactive immune response. Parasite growth depends on the variant specific inherent multiplication rate, drawn from a Normal distribution, and parasite growth is limited by red blood cell availability. Similar to the other models, the innate immune response depends on the current number of parasites, the variant and cross-reactive immune response depend on the variant specific (or group of variants for the cross-reactive immune response) parasites, and the general immune response grows slowly during the course of an infection. Differing to the other models, the four immune responses are all capped by a maximum efficacy level, and the variant specific and cross-reactive immune response depend on the number of available immune cells. The cross-reactive immune response acts on groups of variants randomly grouped and pre-determined. The general immune response, instead of being dependent on time or cumulative parasite density like the other models, here increases each day that the number of parasites are above a certain threshold.

Because the main aim of this model was to understand the key assumptions of the biological mechanisms included in the mechanistic within host model, the model's main advantage compared to the others rely on the sensitivity analysis in the parameter values. Indeed, it is a deterministic model, but a wide range of values for all the parameters have been evaluated using the Latin Hypercube Sampling method. In addition, different switching networks, enabling parasites to switch from one variant to another, were evaluated.

Gurarie *et al.* (15): This is a discrete 2-day time step model. In this model there is a continuous production and loss of uninfected RBCs, which can become infected by the release of merozoites, and parasite replication is inhibited by two immune effectors, innate and adaptive. The merozoite invasion depends on available RBCs so that when the ratio between the number of merozoite and the number of available RBCs becomes large, merozoites start competing for available RBCs. Despite this assumption, it is not clear RBC availability is important for human *P. falciparum* infection. The innate immune response depends on the number of infected cells, activated after reaching a certain parasite density threshold level, and reaching a maximum clearance level. The adaptive immune response is triggered by the product of infected RBCs, and the combined effector pool of the innate and adaptive response, which is a way to allow for adaptive immune memory, and it also depends on an activation threshold. Similar to the other models, the adaptive immune response takes more time to develop but has a slower decay time compared to the innate immune response.

Rather than explicitly modelling the different variant populations, the variant switching dynamics, and variant specific immune response, such as in other models, the model greatly simplifies the equations by using proxies of the more complex underlying mechanisms. First the model only includes innate and adaptive immune responses, making no distinction between the variant specific and general adaptive immune response. Second the parasite population is taken as a whole and does not differentiate between the variant-specific populations. Nevertheless, the variant switching and resulting decrease in an effective adaptive immune response is implicitly accounted for by forcing “random falls” at each replication cycle (where potentially parasites switched to a new variant) of the effective adaptive immune response. To implicitly account for the variant-transcending immune response, the amplitude of the random falls of the effective adaptive immune response decrease in time.

The model was fitted using the malariatherapy data (from 122 patients), and each malariatherapy patient was considered a random realization of a stochastic process. The fitting process was done in two steps to fit the first parasitemia peak assumed deterministic and a second stochastic step for parasitemia post first peak. The model outcome was compared to minimum and maximum of parasite density time-series for each patient, and 50 simulations per patient were selected as an ensemble of best choices (Table S3). The calibration process of the model resulted in a range of parameter values that can be randomly chosen at each realization, and thus allow for variability in the parasite dynamics as observed in the malariatherapy dataset. These parameters relate to the invasion probability, the replication of parasites, immune response efficiencies and activation thresholds, and the assumed antigenically distinct variant clusters.

McKenzie & Bossert (16) : This model is the only model studied here that uses differential equations rather than discrete equations to represent the malaria within host dynamics. The model includes merozoite replication and conversion of parasites into gametocytes and includes the effect of two types of immune responses, innate and adaptive. There is no distinction of variant specific parasites. Both immune responses are stimulated by the asexual density, with a stronger stimulus of the innate effectors, but the dynamic of the innate effectors assume the decay and the removal of the effectors, while the adaptive immune response grows with the asexual parasite density and has no decay or removal in time (note

that the equation for the adaptive effectors include a decay component, but its parameter w = 0 in (16).).

Although not explored in this study, the model tracks different genotypes, includes cross-reactivity, and in addition, investigated several aspect of gametocyte dynamics. Cross-reactivity is included by allowing for immune responses to one genotype to be stimulated by the asexual parasite density of another genotype.

The model was fitted to data from 63 malariatherapy patients who were inoculated with the McLendon strain, including only the parasite densities in time before any drug was administered. The model was fitted to data from each of the 63 patients individually, using the steepest-descent algorithm and comparing model outputs to parasite density time-series (Table S3), and an average of the parameter values was reported and used for their transmission model.

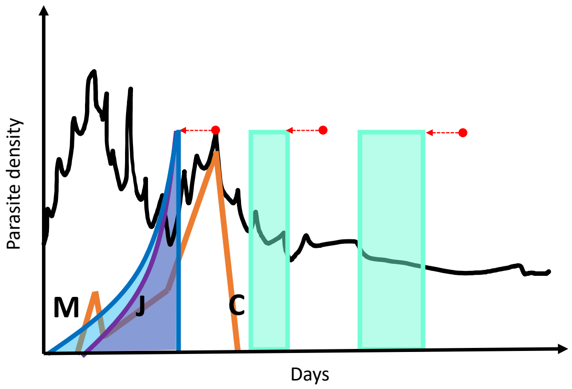

Figure S1: Schematic illustration of an infection and variant specific immune responses at a given time point from models Molineaux et al., Johnston et al., and Challenger et al.. *Example illustration of total parasite density in time (black) with an example of a single variant parasite density in orange. The parasite density affecting the variant specific immune response is represented by the coloured area in blue for Molineaux et al., in purple for Johnston et al., and in green for two examples at two different time points for Challenger et al.. At a given time point, represented by a red dot, the variant specific immune response in the Molineaux et al. (M) and Johnston et al. (J) model depend on the variant specific parasite density (orange) from eight days prior to the time point, and the effect of the parasite density decreases in time, with Molineaux et al. assuming a slower decay rate than Johnston et al.. For Challenger et al. (C), the total parasite density (black) in a given time frame eight days prior to the time point affect the variant specific immune response, with the time frame increasing further along in the infection, implicitly including immune memory, here represented by an example of two time points (red).*

***Table S1: Summary statistics of the observed malariatherapy data and the models**. Summary statistics from the simulation are shown in the first row, and the published results in the second row in italics. Source of published statistics: data [1], Johnston et al.(2), Challenger et al.(4), Gatton & Cheng (5).*

	Data			Molineaux *et al*			Johnston *et al*			Challenger *et al*			Gatton & *Cheng*			Eckhoff		
	med	min	max	med	min	max	med	min	max	med	min	max	med	min	max	med	min	max
**initial slope**	**0.49**	0.19	0.87	**0.50**	0.22	0.74	**0.52**	0.19	0.74	**0.49**	0.15	0.72	**0.47**	0.28	0.56	**0.35**	0.32	0.39
				0.49	*0.19*	*0.87*	***0.52***	*0.24*	*0.75*	***0.49***	*0.25*	*0.71*						
**log10(density) peaks**	**4.79**	3.37	5.66	**4.91**	4.46	5.30	**4.73**	3.55	5.58	**4.66**	3.49	5.56	**4.93**	4.49	5.54	**4.80**	4.76	4.84
				4.79	*3.37*	*5.66*	***4.78***	*3.69*	*5.67*	***4.77***	*3.57*	*5.61*	***4.81***	*4.47*	*5.31*			
**n peaks**	**10.00**	2.00	15.00	**5.83**	3.83	15.49	**9.19**	2.20	16.74	**7.31**	1.20	16.23	**5.86**	1.03	11.66	**6.71**	3.37	14.54
				10.00	*2.00*	*17.00*	***9.00***	*2.00*	*17.00*	***7.70***	*1.70*	*15.50*	***10.00***	*1.00*	*27.00*			
**slope peaks**	**-0.01**	-0.07	0.00	**-0.02**	-0.05	0.00	**-0.02**	-0.08	-0.01	**-0.02**	-0.07	0.00	**-0.01**	-0.03	0.01	**-0.04**	-0.07	-0.02
				-0.01	*-0.07*	*0.00*	***-0.02***	*-0.09*	*-0.01*	***-0.01***	*-0.03*	*0.00*	***-0.01***	*-0.03*	*-0.01*			
**geo. mean peak interval**	**20.48**	14.40	77.77	**16.47**	10.95	39.61	**22.40**	14.19	46.77	**22.41**	12.31	57.67	**20.89**	13.55	31.97	**23.56**	15.51	42.45
				20.00	*14.40*	*77.80*	***14.60***	*1.50*	*28.40*	***22.80***	*12.20*	*61.00*	***22.00***	*14.20*	*29.30*			
**sd peak interval**	**0.20**	0.03	0.47	**0.17**	0.03	0.50	**0.31**	0.10	0.57	**0.21**	0.05	0.38	**0.19**	0.03	0.29	**0.25**	0.08	0.35
				0.20	*0.03*	*0.47*	***0.31***	*0.04*	*0.56*	***0.22***	*0.03*	*0.48*	***0.22***	*0.07*	*0.29*			
**prop. + 1st half**	**0.88**	0.37	1.00	**0.86**	0.25	0.95	**1.00**	0.69	1.00	**1.00**	0.35	1.00	**0.90**	0.64	1.00	**0.75**	0.37	1
				0.88	*0.40*	*1.00*	***0.97***	*0.57*	*1.00*	***0.97***	*0.46*	*1.00*	***0.67***	*0.48*	*1.00*			
**prop. + 2nd half**	**0.45**	0.07	0.93	**0.33**	0.00	0.90	**0.66**	0.12	1.00	**0.99**	0.21	1.00	**0.75**	0.52	1.00	**0.28**	0.09	0.88
				0.46	*0.08*	*0.94*	***0.58***	*0.12*	*1.00*	***0.79***	*0.10*	*1.00*	***0.61***	*0.37*	*1.00*			
**last + day**	**215**	37	405	**142**	74	599	**182**	37	366	**123**	24	257	**129**	20	265	**177**	82	439
				215	*37*	*405*	***193***	*38*	*404*	***198***	*39*	*409*	***239***	*10*	*590*			

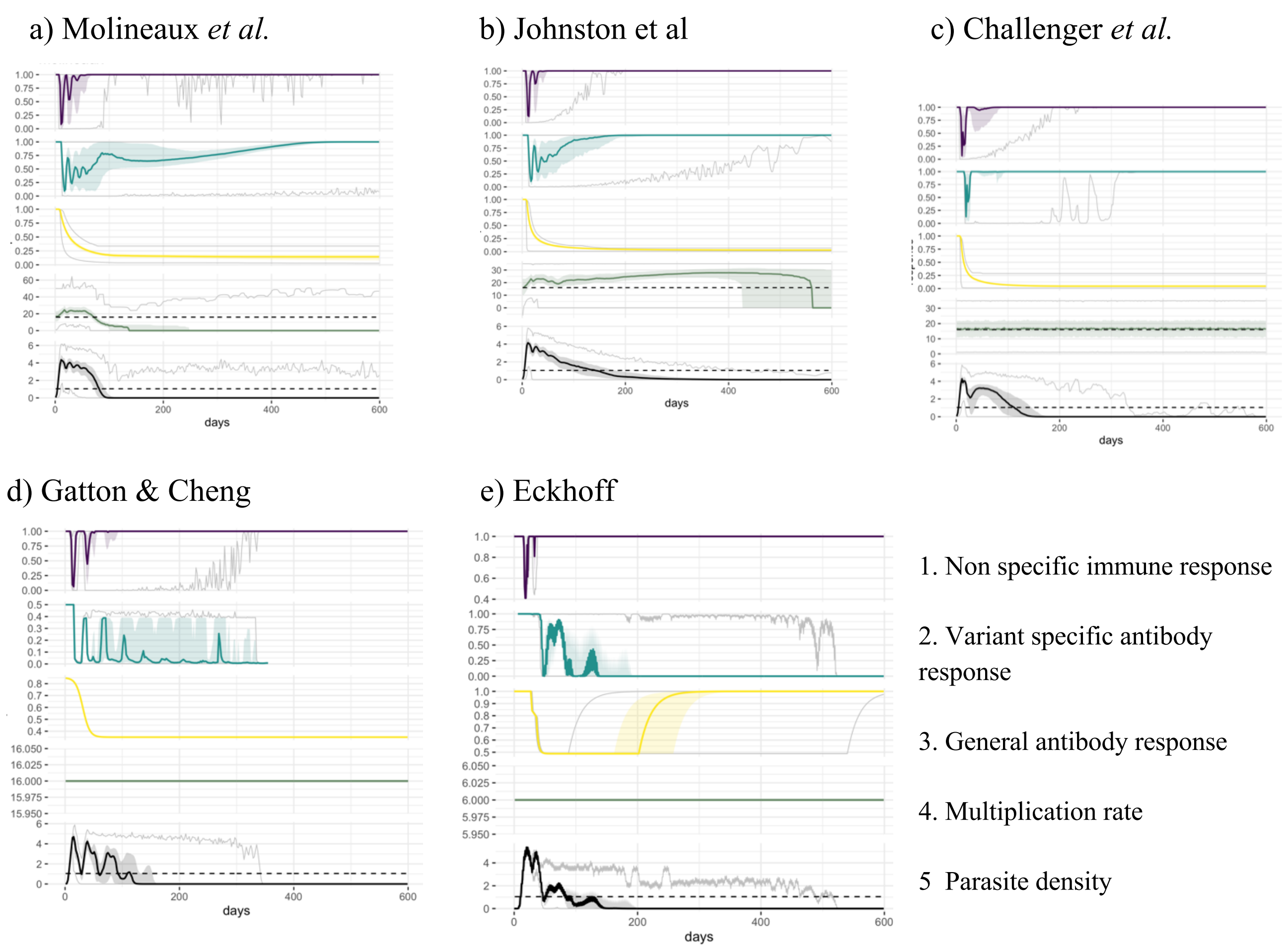

Figure S2: Immune responses, multiplication rates and asexual parasite densities in different models. *Each panel shows, from top to bottom, 1. the effect of the innate immune response, 2. the effect of the variant specific immune response (see methods for calculations), 3. the effect of the general adaptive immune response, 4. the average (across variants, see methods) multiplication rate, and 5. the log10 asexual density. X-axis represents days of infection, solid line the median, shaded area the interquartile range (Q25-Q75) and the gray lines the minimum and maximum resulting from the 1750 simulations. The horizontal dashed green line represents an average multiplication rate of 16, the horizontal dashed black line indicate the threshold of positive density (10PBRC/µl). Results are shown for* ***a)*** *Molineaux et al.,* ***b)*** *Johnston et al., and* ***c)*** *Challenger et al.,* ***d)*** *Gatton & Cheng, and* ***e)*** *Eckhoff*

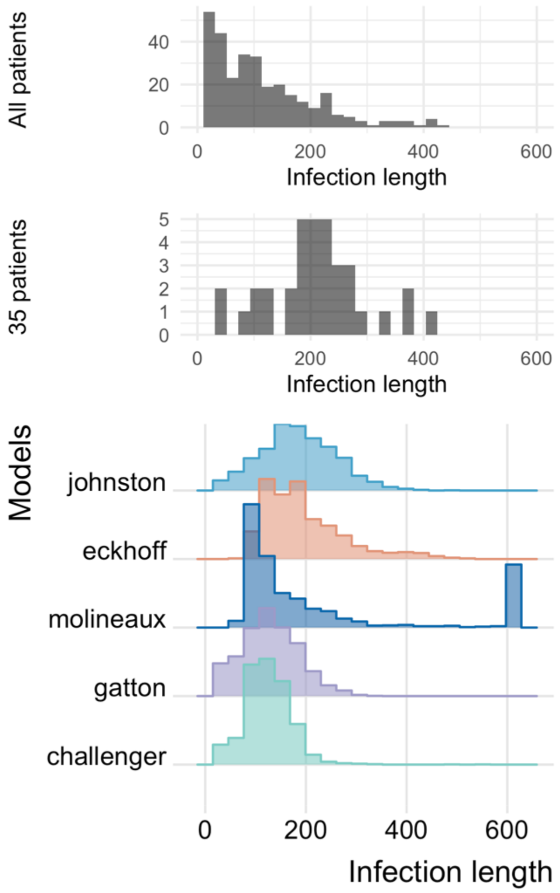

Figure S3: Distribution of infection length in the malariatherapy dataset and in the models*. From top to bottom, the entire malariatherapy dataset as described by Collins et al (n=316 patients), the 35 malariatherapy records used for the Molineaux model, and the 5 models. Simulation time was set to 600 days, thus infections which did not end at simulation end appear a peak at day 600.*

Table S2: Model equations

<table>
<tr><th colspan="3">Within-host mechanistic models</th></tr>
<tr><td>Molineaux et al. (1) and Molineaux adapted models (2,4)</td><td>Gatton & Cheng model (5)</td><td>Eckhoff (9)</td></tr>
<tr><td colspan="3">Parasite dynamics</td></tr>
<tr>
<td>Parasite replication, influenced by immune responses, and antigenic variation

• Molineaux et al. and Johnston et al.

$P_i(t+2)' = \left((1-s)P_i(t) + sp_{i(t)}\sum_{j=1}^{v} P_j(t)\right) m_i S_c(t) S_i(t) S_m(t)$

• Challenger et al.

$P(t+2) = m(t)S_c(t)S_v(t)S_m(t)P(t)$

Parasite density

• Molineaux et al. and Johnston et al.

$P_c(t) = \sum_{i=1}^{v} P_i(t)$
$P_i(t+2) = \begin{cases} P_i(t+2)' & if\ \ P_i(t+2)' \geq 10^{-5} \\ 0 & otherwise \end{cases}$</td>
<td>Number of parasites

$X_t = \sum_{i=1}^{50} X_{t,i}$

$X_t = \begin{cases} X_t & if\ \ X_t \geq 1 \\ 0 & otherwise \end{cases}$

Parasite antigenic variation

$X_{t,i} = X_{t,i} + st_{t,i} - sw_{t,i}$

Parasite replication, influenced by immune responses

$X_{t+1,i} = \text{Bi}(16X_{t,j}, (1-m_t)/Ab_{\text{t,i}})$

• If $X_t > NSI\ threshold\ (T)$:

$X_{t,i} = \begin{cases} Bi\left(X_{t,i}, \exp\left(-k * \frac{X_t - T}{T}\right)\right) if\ k(X_t - T) \leq 10 \\ Bi\left(X_{t,i}, \exp(-10)\right) if\ k(X_t - T) > 10 \end{cases}$</td>
<td>$t_{asexual} = T_{asexual}$, and $t_{asexual,n+1} = t_{asexual,n} - \Delta t$ defines a 48h timer for including both continuous and discrete events.

Concentration per microliter of parasites expressing antigen i:

$X_i = N_i/(number\ of\ microliters\ of\ blood)$

Number of IRBCs of antigen n created by previous generation expressing antigen n

$N_n^1 = \left((1-k_{gametocyte})N_n - \sum^{nantigenswitch} N_{n+1,n}\right) N_{IRBCmerozoites} Z_{MS}$

For all n, i=1 to $n_{antigenswitch}$, add in switching IRBCs

$N_{n+i}^1 = N_{n+i,n} N_{IRBCmerozoites} Z_{MS}$

Number of IRBCs expressing antigen i whose merozoites will create IRBCs in the next generation n+1 expressing same antigen i

$N_{i,n+1} = N_{i,n} - Binomial(N_{i,n}, P_{kill,i})$

$P_{kill,i} = 1 - \exp\left(-\Delta t\left(\frac{C_{innate}Y_{fever}}{1+Y_{fever}} + C_{antibody}\left(Y_{antibody,i} + Y_{Minorepitope,i}c_{MinorMod}\right)\right)\right)$</td>
</tr>
<tr><td colspan="3">Antigenic switch</td></tr>
</table>

• *Molineaux et al.and Johnston et al*<br>$p_i(t) = \begin{cases} 0 & if \;\; S_i(t) < 0.1 \\ \frac{q^i S_i(t)}{\sum_{j=1}^{v} q^j S_j(t)} & otherwise \end{cases}$	$st_{t,i} = \sum_{j=1, j \neq i}^{50} Bi(X_{t,j}, 0.18\mathrm{p_i} / \sum_{i=1}^{50} p_i)$<br>$sw_{t,i} = \sum_{j=1, j \neq i}^{50} Bi(X_{t,i}, 0.18\mathrm{p_j} / \sum_{i=1}^{50} p_i)$<br>$p_i = \begin{cases} 0 \; for \; var \; 1 \\ 1 \; for \; var \; 2 \\ \frac{p_{i-1}}{1.3} \; for \; var \; 3 \; to \; c \\ \frac{p_{i-1}}{1 * 10^7} \; for \; var \; (c+1) \\ \frac{p_{i-1}}{1.18} \; for \; var \; (c+2) \; to \; 50 \end{cases}$	*Number of IRBCs of antigen n switching to express antigen n+i*<br>$N_{n+i,n} = Poisson(K_{antigen} N_n), 1 \leq i \leq n_{antigenswitch}$
**Immune responses**		
innate<br>$S_c(t) = \left(1 + \left(\frac{P_c(t)}{P_c^*}\right)^{\kappa_c}\right)^{-1}$<br>*variant specific*<br>• Molineaux *et al.*and Johnston *et al*<br>$S_i(t) = \left(1 + \left(\frac{\sum_{\tau=0}^{t-\delta_v} P_i(\tau) e^{-\sigma(t-\tau-\delta_v)}}{P_v^*}\right)^{\kappa_v}\right)^{-1}$<br>• Challenger *et al*<br>$S_v(t) = \left(1 + \left(\frac{\sum_{\tau=f(t)}^{t-8} P(\tau)}{P_{v2}^*}\right)^{\kappa_v}\right)^{-1}$	*innate*<br>$T = \begin{cases} 1/SF * 10^{0.271t+1.6864+N(0,0.3)} \; if \; t \leq 3 \\ 1/SF * 10^{3.39+N(0,0.3)} \; if \; t > 3 \end{cases}$<br>*anti-PfEMP1*<br>• If $X_{t,i} \geq 6 * 10^7$ for the first time in the last 20 days<br>• Trigger antibody for variant *i*<br>$s_i = t + 4$<br>$A_i = \begin{cases} N(\alpha, 100) & for \; the \; first \; Ab \; produced \\ N(\alpha + 200, 100) & otherwise \end{cases}$<br>$B_i = N(\psi, 0.07)$<br>• Calculate amount of antibody present for each variant	*innate*<br>$\begin{cases} for \;\; t_{asexual} > 0: \frac{dY_{innate}}{dt} = \frac{1}{\tau_{innate}} \frac{\sum_i X_i(1 - Y_{antibody,i})}{\sum_i X_i(1 - Y_{antibody,i}) + X_{50,innate}} \\ for \; t_{asexual} = 0: \; \Delta Y_{innate} = \frac{\sum X_{schizont}}{\sum X_{schizont} + X_{50,innate}} \end{cases}$<br>$Y_{fever} = k_{fever} Y_{innate}$<br>*variant specific*<br>For i=1 to 50, if $X_i > 0$<br>$\frac{dY_{capacity,i}}{dt} = \begin{cases} \frac{k_{pfemporminor}}{\tau_{capacity}} \frac{X_i + k_{antibody,min} X_{50,antibody}}{X_i + X_{50,antibody}} (1 - Y_{capacity,i}), Y_{capacity,i} < 0.4 \\ \frac{1}{\tau_{hyperimmunity}} (1 - Y_{capacity,i}), Y_{capacity,i} \geq 0.4 \end{cases}$<br>$k_{pfemporminor} = \begin{cases} 1 \; for \; PfEMP1 \\ k_{minormod} \; for \; minor \; epitopes \end{cases}$

Variant transcending<br>$$S_m(t) = (1-\beta)\left(1+\left(\frac{\sum_{\tau=0}^{t-\delta_m}\hat{P}_c(\tau)e^{-\rho(t-\tau-\delta_m)}}{P_v^*}\right)^{\kappa_m}\right)^{-1} + \beta$$<br>$$\hat{P}_c(t) = \begin{cases} P_c(t) & if\ P_c(t) < C \\ C & otherwise \end{cases}$$<br>$$P_c^*(t) = k_c * (first\ local\ max.density)$$<br>$$P_m^*(t) = k_m * [(last\ pos.day) - (first\ pos.day)]$$	$$Ab_{t,i} = \begin{cases} A_i z_i e_i^{-Bz_i} & if\ t > s_i \\ 1\ if\ t \le s_i\ and\ Ab_{t,j} = 1\ for\ all\ j \\ 1.05\ if\ t \le s_i\ and\ Ab_{t,j} > 1\ for\ any\ j \end{cases}$$<br>$$z_i = (2t - s_i)/30$$<br>*General adaptive*<br>$$m_t = m_0 + 0.5/(1+\exp(-(2t-30)/6))$$	$$\frac{dY_{antibody,i}}{dt} = \frac{1}{\tau_{abprod}}(Y_{capacity,i} - Y_{antibody,i}), Y_{capacity,i} > 0.3$$<br>For i=1 to 50, if $X_i = 0$<br>$$\frac{dY_{capacity,i}}{dt} = -\frac{1}{\tau_{capdecay}}(Y_{capacity,i} - Y_{memory}), Y_{capacity,i} > Y_{memory}$$<br>$$\frac{dY_{antibody,i}}{dt} = -\frac{1}{\tau_{abdecay}}Y_{antibody,i}$$<br>*Anti-MSP*<br>$$if\ t_{asexual} = 0$$<br>$$\Delta Y_{MSP} = K_{MSP}\frac{\sum X_{schizont}}{\sum X_{schizont} + X_{50,antibody}}(1 - Y_{MSP})$$<br>$$Z_{MS} = (1 - C_{merozoite}Y_{antibody,MSP})$$
**Variables**		
$P_i(t)$ = density of variant *i,* as PRBC/µl blood<br><br>$P_c(t)$ = total parasite density, as PRBC/µl blood<br><br>• Molineaux *et al.* and Johnston *et al.*<br><br>$p_i(t)$ = probability that a switching PBRC switches to variant *i*<br><br>$S_c(t), S_i(t), S_m(t)$ = probability that a parasite of variant *i* escapes control by the innate immune response, acquired variant-specific immune response, and acquired variant-transcending immune response, respectively, in the interval (t,t+2)	$X_{t,i}$ = number of parasites of variant *i*<br><br>X_t = total number of parasites<br><br>$st_{t,i}$= number of parasites switching to variant *i*<br><br>$sw_{t,i}$= number of parasites switching from variant *i*<br><br>p_i = switch rate<br><br>T , $Ab_{t,i}$, m_t = innate immunity, anti-PfEMP1 antibodies ,clonal immunity	N_n = number of IRBCs expressing antigen n<br><br>$N_{n,n+i}$ = number of IRBCs expressing antigen n whose merozoites will create IRBC's in the next generation expressing antigen n+i<br><br>N_n^1 = number of IRBCs in the new asexual cycle expressing antigen i<br><br>X_i = Concentration per microliter of parasites expressing antigen i<br><br>Y_{innate} = Level of pro-inflammatory cytokines<br><br>Y_{fever} = Level of fever due to pro-inflammatory cytokines

• Challenger *et al*<br><br>$S_c(t), S_v(t), S_m(t)$ = probability that a parasite escapes control by the innate immune response, acquired variant-specific immune response, and acquired variant-transcending immune response, respectively, in the interval (t,t+2)		$Y_{antibody,i}$ = Level of antibodies specific to antigen *i*<br><br>$Y_{capacity,i}$ = Variable representing changing capacity to produce antibodies specific to antigen *i*
**Parameters**		
v = number of variants per clone<br><br>s = fraction of parasites switching among variants per two-day<br><br>q = parameter of the geometric distribution of switching probabilites<br><br>P_c^*, P_m^* = two host-specific critical densities<br><br>k_c, k_m = two constants allowing calculation of P_c^*, P_m^* from host specific data<br><br>P_v^* = critical density of a variant, common to all variants<br><br>$\kappa_c, \kappa_v, \kappa_m$ = stiffness parameters for a saturation of innate immune response, acquired variant-specific immune response, and acquired variant-transcending immune response<br><br>C = maximum daily antigenic stimulus, per µl, of the acquired variant-transcending immune response<br><br>σ = decay parameters, per day, of the acquired variant-specific immune response<br><br>δ_v, δ_m = delay parameters, in days, of the acquired variant-specific and variant transcending immune response, respectively<br><br>β = maximum value of S_m	Switching parameters:<br><br>• c; number of 'fast' switching *var* genes<br><br>Non specific immunity<br><br>• SF, scaling factor correcting for difference between onset of NSI and pyrogenic threshold<br>• k, feedback constant for sensitivity of parasites to NSI response<br><br>Specific immunity<br><br>• m_0 probability of parasites being killed by specific immunity at start of blood-stage infection<br><br>Anti-PfEMP1 immunity<br><br>• α, parameter influencing magnitude of antibody response<br>• Ψ (months[-1]), reciprocal of time to antibody peak	Immunological:<br><br>$X_{50,innate}$ = parasitemia at which innate immune response is half of maximum<br><br>$X_{50,capacity}$ = parasitemia at which antibody immune response is half of maximum<br><br>τ_{innate} = inverse of maximum immune response rate<br><br>$\tau_{capacity}$ = inverse of maximum antibody response rate<br><br>$\tau_{hyperimmunity}$ = inverse of development of hyperimmunity<br><br>$k_{antibodymin}$ = minimum antibody response to antigen<br><br>$k_{minormod}$ = modification of response rate for minor epitopes<br><br>C_{innate} = rate at which innate immune response kills IRBCs<br><br>$C_{antibody}$ = rate at which specific antibody immune response kills IRBCs<br><br>$C_{minormod}$ = modification of antibody kill rate for minor epitopes<br><br>$C_{merozoite}$ = maximum blocking of merozoite invasion by MSP antibodies<br><br>K_{MSP} = per cycle growth rate of MSP antibody response

$m_i \sim N(\mu_m, \sigma_m^2)$ *truncated to ensure* $m_i \geq 1$ (*Molineaux et al*) *or* $35 \geq m_i \geq 1$ (*Johnston et al*) : basic multiplication factor, per two-day cycle, of variant *i;*<br><br>$m \sim N(\mu, \sigma), \frac{\langle (m(t)-\mu)(m(t-\theta)-\mu) \rangle}{\sigma^2} = g^{\theta}, 0 < g < 1$: average growth rate at time t		Y_{memory} = immunological memory level<br><br>$T_{hyperimmunity}$ = duration of hyperimmunity after removal of antigen<br><br>Parasitological:<br><br>$N_{IRBCmerozoites}$ = Merozoites per IRBC schizont<br><br>$T_{asexual}$ = Duration of asexual cycle<br><br>$N_{variants}$ = Number of PfEMP-1 variants per clone<br><br>$N_{minorepitopes}$ = Number of minor epitopes per clone<br><br>$K_{antigen}$ = PfEMP-1 switch rate<br><br>$n_{antigenswitch}$ = number of non-suppressed available variants per IRBC
Comparing immune response effects between Molineaux *et al.* and *Molineaux adapted* models with Gatton *et al.* and Eckhoff		
• *effect of the innate immune response*		
S_c	$\exp\left(-\frac{k(X_t - T)}{T}\right)$	$\exp\left(-C_{innate}\frac{k_{fever}Y_{innate}}{1 + k_{fever}Y_{innate}}\right)$
• *effect of the variant specific immune response*		
$S_{v,i}$	$1/Ab_{t,i}$	$\exp(-C_{antibody}(Y_{antibody} + Y_{minorepitope}\, c_{MinorMod}))$
• *effect of the general adaptive immune response*		
S_m	*1-m(t)*	$Z_{MS} = (1 - C_{merozoite}\, Y_{antibody,MSP})$
**Within-host mechanistic models**		
Childs & Buckee (14)	Gurarie *et al (15)*	McKenzie & Bossert (16)
Parasite dynamics		

Number of parasites, dependent on growth and immune responses<br><br>$P_i(t+1) = g_i(t+1)I(t+1)\Gamma_{VS_i}(t+1)\Gamma_{CR_i}(t+1)M(t+1),$<br><br>*Parasite growth, dependent on intrinsic growth rate and inherent switching between variants, limited by red blood cells availability*<br><br>$g_i(t+1) = \left(1-\exp\left(-\frac{K(t)}{\sum_j p_j(t)}\right)\right)\left[\gamma_i p_i(t)(1-\omega_i) + \omega_i \sum_{j\neq i} \gamma_j \beta_{ji} p_j(t)\right]$	*Uninfected red blood cells, depend on homeostatic production and loss of red blood cells, and the loss due to merozoite invasion*<br><br>$x' = (1-\delta)x_0 + \delta x - \rho\left(\frac{M}{x}\right)x$<br><br>*Infected red blood cells, depend on combined level of innate and adaptive immune response, and on merozoite invasion*<br><br>$y' = 2^{-(a+b)}\rho\left(\frac{M}{x}\right)x$<br><br>*Merozoite equation depends on the effective replication rate of the parasite:* $M = ry + S$<br><br>*Effective probability of invasion decreases according to:*<br><br>$\rho(z) = 1 - e^{-z}$<br><br>$z = M/x$	*Asexual parasite density, depend on daily replication of asexual parasite, daily conversion into gametocytes, and the effect of immune response:*<br><br>$\frac{dM_u}{dt} = \left(a_u - z_u - c_u I - \Sigma b_{u,v} J_v\right) M_u$
**Antigenic switch**		
$g_i(t+1) \propto \omega_i \sum_{j\neq i} \gamma_j \beta_{ji} p_j(t)$	-	-
**Immune responses**		
innate<br><br>$I(t+1) = \min\left(E_I, \left(\frac{1+\sum_i p_i(t)}{1+\sum_i p_i(t) + C_I}\right) E_I\right)$<br><br>*variant specific*<br><br>• *Dynamics of immune cells, depend on pre-existing cells, expansion when variant specific parasites in large quantities, decay, and restricted by total number of immune cells available*	*Innate effectors, stimulation triggered by parasite level relative to threshold A, and has a short life span*<br><br>$a' = f_a \phi_a\left(\frac{y}{A}\right) + \sigma_a a$<br><br>*Adaptive effectors, stimulation triggered by parasite level relative to threshold B, and has a longer life span*<br><br>$b' = q f_b \phi_b\left(\frac{y(a+b)}{B}\right) + \sigma_b b$	*innate immune effectors*<br><br>$\frac{dI}{dt} = \Sigma s_v M_v - I\Sigma k_v M_v - qI$<br><br>*acquired immune effectors:*<br><br>$\frac{dJ_u}{dt} = \Sigma r_{u,v} M_v - w_u J_u$

$VS_i(t+1) = VS_i(t)\left(1+\pi_{VS}\exp\left(-\frac{\psi_{VS}}{p_i(t-\tau)+1}\right)\right)\left(1 - \mu_{VS}\left(1-\exp\left(-\frac{\psi_{VS}}{p_i(t)+1}\right)\right)\right)\left(1 - \exp\left(-\frac{K_{Imm}}{\sum_j VS_j(t)}\right)\right)$<br><br>• *Effectiveness of response, depend on relative amount of parasite variants and specific immune cells*<br><br>$\Gamma_{VS_i}(t+1) = E_{VS}\left(1-\exp\left(-\frac{1+g_i(t)}{VS_i(t)}\right)\right)$<br><br>*cross-reactive:*<br><br>• *Dynamics of immune cells, depend on pre-existing cells, expansion when group of variant specific parasites in large quantities, decay, and restricted by total number of immune cells available*<br><br>$CR_i(t+1) = CR_i(t)\left(1+\pi_{CR}\exp\left(-\frac{\psi_{CR}}{1+\sum_{j\sim i} p_i(t-\tau)}\right)\right)\left(1 - \mu_{CR}\left(1-\exp\left(-\frac{\psi_{CR}}{1+\sum_{j\sim i} p_i(t)}\right)\right)\right)\left(1 - \exp\left(-\frac{K_{Imm}}{\sum_j CR_j(t)}\right)\right)$<br><br>• *Effectiveness of response, depend on relative amount of a group of parasite variants and specific immune cells*<br><br>$\Gamma_{CR_i}(t+1) = E_{CR}\left(1-\exp\left(-\frac{1+\sum_{j\sim i} g_i(t)}{CR_i(t)}\right)\right)$<br><br>*Variant transcending*	• *Antigenic variation accounted for by random falls in effector, q_0^t an exponential function with base:*<br><br>$q_0 = 2^{-1/m} < 1$	

$M(t+1) = \min\left(E_M, \frac{1+\sum_t \chi_t}{1+C_M+\sum_t \chi_t} E_M\right)$<br><br>$\chi_t = \begin{cases} 1, & if \sum_i p_i(t) > 10^7 \\ 0 & otherwise \end{cases}$		
**Variables**		
$P_i(t)$ = overall number of parasites of the *i*th antigenic variant<br><br>$g_1(t)$ = parasite growth, in the absence of any immune response<br><br>$I(t), \Gamma_{VS_i}, \Gamma_{CR_i}, M(t)$ = effectiveness of the innate, variant specific, cross-reactive, and general immune response, respectively<br><br>CR_i, VS_i= dynamics of the variant specific, and cross-reactive immune cells, respectively.	x = uninfected RBC measured per microliter of blood<br><br>y = infected RBC measured per microliter of blood<br><br>M = number of merozoites released (a result of y(t))<br><br>a,b = dimensionless variables representing innate and adaptive immune effectors, respectively	M = asexual forms<br><br>J = acquired-immunity effectors<br><br>I = innate-immunity effectors<br><br>subscript v : different genotypes
**Parameters**		
Immunological:<br><br>E_I, E_{VS}, E_{CR}, E_M = maximum efficacy of innate, variant specific, cross-reactive, and general immune response, respectively<br><br>$C_I, \psi_{VS}, \psi_{CR}, C_M$ = half-maximal activation of innate, variant specific, cross-reactive, and general immune response, respectively<br><br>$K(0), K_{Imm}$= restriction of total number of red blood cells, and immune cells, respectively<br><br>μ_{VS}, μ_{CR} = maximum decay rate of variant-specific, and cross-reactive immune cells, respectively<br><br>π_{VS}, π_{CR} = maximum growth rate of variant-specific, and cross-reactive immune cells, respectively	Immunological:<br><br>A,B = Threshold to trigger innate and adaptive immune effectors, respectively<br><br>ϕ_a, ϕ_b= Hill function defining innate and adaptive immune stimulation, respectively<br><br>f_a, f_b= maximal stimulation efficiency of innate and adaptive immune response, respectively<br><br>$a_M = \frac{f_a}{1-\sigma_a}, b_M = \frac{f_b}{1-\sigma_b}$ = maximal clearance levels of innate and adaptive immune effectors, respectively. $a_M + b_M \gg \log(r)$<br><br>δ_a, δ_b = survival rate per cycle of innate and adaptive effectors, respectively	Immunological:<br><br>c, b = daily removal rate of asexual forms by innate-immune, and acquired immune response effectors, respectively<br><br>s, r = rate of stimulation of the innate immune, and acquired immune response (effectors per asexual form)<br><br>k = removal rate of innate-immune effectors (effectors per effector asexual form)<br><br>q, w = decay rate of innate-immune, and acquired immune response effectors, respectively<br><br>Parasitological:<br><br>a = daily replication rate of asexual blood forms,

τ = delay of adaptive immune response activation (days)<br><br>Parasitological:<br><br>γ_i = intrinsic growth rate of variant *i*<br><br>ω_i = percentage switching to a new variant from variant *i*<br><br>β_{ji} = switching probability from variant *i* to variant *j*	Parasitological:<br><br>*r* = effective replication rate<br><br>*p* = probability of a merozoite to invade an uninfected RBC<br><br>*m* = number of antigenically distinct variant clusters<br><br>x_0 = normal concentration of uninfected RBC	*z* = conversion rate of asexual forms to gametocytes

Table S3: Model fitting, *as described in the initial publications. Fitting methods and data might have been modified subsequently.*

**Models**	**Molineaux *et al.***	**Johnston *et al.***	**Challenger *et al.***	**Gatton *et al.***	**Childs & Buckee.**	**Eckhoff**	**McKenzie & Bossert**	**Gurarie *et al.***
Fitting method	Informed trial and error (no systematic fitting) to find the best set of parameters. In a second step each patient was simulated 50 times (with different multiplication factors), and χ^2 was used to identify best patient specific match	Bootstrapping, not all parameter space explored. The model was run 1,000 times and 50 samples of 50 runs each were selected.<br><br>Goodness-of-fit, was assessed with relative errors between model outputs tested outcome	Same than Johnston *et al*, but with MCMC using Metropolis-Hasting algorithm to generate random walks in parameter space	Fitted to the patient data using sets of 100 simulations, altering the 6 parameters until the distribution of simulations did not differ significantly to that of the patient data.	Stochastic sampling of parameters using a Latin Hypercube Sampling. Results include a minimum of 100 000 replicates. This model was not fitted but the range of parameter values were defined according to literature	Extensive simulations and ensembles of simulated infection trajectories generated for ranges of combinations consistent with observed infection behaviors.	Fitted the model to each of the 63 individual charts using a steepest-descent algorithm to determine the parameter set that minimized the sum of squared deviations. Starting values based on the literature and their previous work.	Two steps: a deterministic fit to the first wave of parasitemia, and a second stochastic step for parasitemia patterns following the initial wave. Based on these steps 'best choices' of in-host parameters are selected, by defining min/max ensemble envelop' by minimizing its

								distance from the ensemble mean curve
Outcome tested for the fitting	distribution of durations of infection from malariatherapy as well as the minimum, median, and maximum of the 9 summary statistics			max parasitemia, number of days with fever, number of days with parasitemia higher than 10 per microliter, number of days with parasitemia higher than 10000 per microliter		first parasite density peak, lower secondary peaks, and even lower peaks after 100 days, the possibility of reinfection with homologous strains, the interval between peaks, and the distribution of measured durations.	asexual parasite density and gametocyte parasite density over time for each patient chart	asexual parasite density over time for each patient chart
Fitted parameters	$q, k_c, k_m, P_v^*, \kappa_c, \kappa_v, \kappa_m$ $C, \sigma, \delta_v, \delta_m, \beta$	$\kappa_c, \sigma, P_C^*, P_m^*$	$\sigma, g, P_v^*, \kappa_m, f(t)$	$c, SF, k, m_0, \alpha, \Psi$	All parameter values are explored within ranges	Ranges of several parameters ($X_{50,innate}$, C_{innate}, $C_{antibody}$, $C_{minormod}$, $C_{merozoite}$, K_{MSP}, Y_{memory}, $K_{antigen}$) are investigated	a-z, zy, p, s, c, k, q, b, r	A, B, p, f_a, f_b, m

References

1. Molineaux L, Diebner HH, Eichner M, Collins WE, Jeffery GM, Dietz K. *Plasmodium falciparum* parasitaemia described by a new mathematical model. Parasitology. 2001 Apr;122(4):379–91.

2. Johnston GL, Smith DL, Fidock DA. Malaria's Missing Number: Calculating the Human Component of R0 by a Within-Host Mechanistic Model of Plasmodium falciparum Infection and Transmission. Antia R, editor. PLoS Comput Biol. 2013 Apr 18;9(4):e1003025.

3. Sama W, Dietz K, Smith T. Distribution of survival times of deliberate Plasmodium falciparum infections in tertiary syphilis patients. Trans R Soc Trop Med Hyg. 2006 Sep;100(9):811–6.

4. Challenger JD, Bruxvoort K, Ghani AC, Okell LC. Assessing the impact of imperfect adherence to artemether-lumefantrine on malaria treatment outcomes using within-host modelling. Nat Commun. 2017 Dec;8(1):1373.

5. Gatton ML, Cheng Q. Investigating antigenic variation and other parasitehost interactions in Plasmodium falciparum infections in nave hosts. Parasitology. 2004 Apr;128(4):367–76.

6. Paget-Mcnicol S, Gatton M, Hastings I, Saul A. The *Plasmodium falciparum var* gene switching rate, switching mechanism and patterns of parasite recrudescence described by mathematical modelling. Parasitology. 2002 Mar;124(3):225–35.

7. Gatton ML, Peters JM, Fowler EV, Cheng Q. Switching rates of Plasmodium falciparum var genes: faster than we thought? Trends Parasitol. 2003 May;19(5):202–8.

8. Peters J, Fowler E, Gatton M, Chen N, Saul A, Cheng Q. High diversity and rapid changeover of expressed var genes during the acute phase of Plasmodium falciparum infections in human volunteers. Proc Natl Acad Sci. 2002 Aug 6;99(16):10689–94.

9. Eckhoff P. P. falciparum Infection Durations and Infectiousness Are Shaped by Antigenic Variation and Innate and Adaptive Host Immunity in a Mathematical Model. Snounou G, editor. PLoS ONE. 2012 Sep 19;7(9):e44950.

10. Malaria infection and immune model — Malaria Model documentation [Internet]. [cited 2020 Nov 2]. Available from: https://docs.idmod.org/projects/emod-malaria/en/latest/malaria-model-infection-immunity.html

11. Selvaraj P, Wenger EA, Gerardin J. Seasonality and heterogeneity of malaria transmission determine success of interventions in high-endemic settings: a modeling study. BMC Infect Dis. 2018 Aug 22;18(1):413.

12. Gerardin J, Ouédraogo AL, McCarthy KA, Eckhoff PA, Wenger EA. Characterization of the infectious reservoir of malaria with an agent-based

model calibrated to age-stratified parasite densities and infectiousness. Malar J. 2015 Jun 3;14(1):231.

13. McCarthy KA, Wenger EA, Huynh GH, Eckhoff PA. Calibration of an intrahost malaria model and parameter ensemble evaluation of a pre-erythrocytic vaccine. Malar J. 2015 Jan 7;14(1):6.

14. Childs LM, Buckee CO. Dissecting the determinants of malaria chronicity: why within-host models struggle to reproduce infection dynamics. J R Soc Interface. 2015 Mar;12(104):20141379.

15. Gurarie D, Karl S, Zimmerman PA, King CH, St. Pierre TG, Davis TME. Mathematical Modeling of Malaria Infection with Innate and Adaptive Immunity in Individuals and Agent-Based Communities. Paul RE, editor. PLoS ONE. 2012 Mar 28;7(3):e34040.

16. McKenzie FE, Bossert WH. An integrated model of Plasmodium falciparum dynamics. J Theor Biol. 2005 Feb;232(3):411–26.
